# Molecular characterization of metallo-*β*-lactamase- producing carbapenem-resistant *Enterobacter cloacae* complex isolated in Heilongjiang Province of China

**DOI:** 10.1186/s12879-020-4768-7

**Published:** 2020-01-31

**Authors:** Yongxin Zhao, Jisheng Zhang, Yanjun Fu, Chunjiang Li, Kewang Hu, Shanshan Su, Lan Yu, Yuhang Guo, Yu Fu, Xiaoli Zhang

**Affiliations:** 1 Yongchuan Hospital of Chongqing Medical University, Chongqing, China; 2 the First Affiliated Hospital of Jiamusi University, Jiamusi, Heilongjiang China; 3 Jiamusi University, Jiamusi, Heilongjiang China; 4 Center for Disease Control and Prevention, Jiamusi, Heilongjiang China

**Keywords:** *Enterobacter cloacae* complex, Metallo-*β*-lactamase, Fluoroquinolone resistance, ST93, CREC

## Abstract

**Background:**

*Enterobacter cloacae* complex (ECC) is one of the most common extended-spectrum β-lactamase and carbapenemase-producing pathogen that threatens millions of the elderly and vulnerable sick persons. The objective of this study was to perform the molecular characteristics of the carbapenem-resistant *E. cloacae* complex (CREC) emerged in Heilongjiang Province of China.

**Methods:**

Six CREC strains were isolated from the patients with infectious diseases. The identities of ECC isolates were confirmed by sequencing the polymerase chain reaction (PCR) products of *16S rRNA* gene. The characterization of the CREC isolates were analyzed by sequencing PCR products of the *carbapenemase*, *ampC* and *fluoroquinolone* resistance genes and performing multilocus sequence typing (MLST), pulsed-field gel electrophoresis (PFGE) and whole genome sequencing.

**Results:**

All 6 isolates harbored multiple resistance genes. Of them, 5 carried *metallo-β-lactamases* and one was *bla*_KPC-2_-positive. The levofloxacin and ciprofloxacin-resistant strains had substitutions of *gyrA*83, *gyrA*87, and *parC*80 in the quinolone-resistance determining regions. The MLST analyses revealed that 6 isolates belonged to five sequence types (ST520, ST528, ST1119, ST1120, and ST93) while the PFGE patterns of the isolates fallen into four clusters. The strain ST1120 was found to carry two separated plasmids that encode *bla*_NDM-1_ and *bla*_IMP-4_.

**Conclusions:**

Our study, for the first time, identified a CREC strain that co-produces *bla*_NDM-1_ and *bla*_IMP-4_ in the Northeast China. Our finding emphasizes an urgent need for more intensive surveillance and precaution measures to prevent the CERC spread.

## Background

The carbapenem-resistant *Enterobacteriaceae* (CRE) may cause serious health problems for the elderly and sick persons because the germ has become resistant to most of antibiotics. Among all Enterobacteriaceae, *Enterobacter cloacae* complex (ECC) ranks the third in its ability to cause infections in wounds, urinary tract, intra-abdominal foci, blood, lungs, skin, and other soft tissues [1]. In clinics, Carbapenems are used as a last resort to treat severe Gram-negative bacterial infections when other antibiotics have failed. However, some strains of ECC could develop Carbapenem-resistance through the mechanisms by which the bacteria either constitutively overexpress AmpC β-lactamases and make mutations that reduce its membrane permeability or enhance efflux of antibiotics, or, more commonly, they gain a plasmid that encodes carbapenemase genes to degrade the antibiotics. While the membrane defect associated resistance may be disseminated via clonal expansion, bacteria carrying cabapenemase genes can be spread among different bacterial strains and species by horizontal transfer of large conjugative plasmids. The significance of the global spread of carbapenemase-producing ECC isolates through patient transfer or colonized travelers in Europe, North America, the Far East and Australia has been well described in two reviews [2, 3]. Recent meta-analysis and multicenter clinical study demonstrated a more than two-fold increased risk of mortality in patients infected with carbapenem-resistant gram-negative, MDR-positive bacteria, and a significant increase in healthcare cost [4, 5]. It has been reported that mortality rate in the patients with CRE infections was as high as 44% [6]. The global spread of drug-resistant bacteria also has a significant impact on the environment and on wild animals and livestock since they can acquire MDR bacteria in hospitals and other waste sources [7]. Because multiple mechanisms are involved in the development of drug-resistance, accurate identification of species and subspecies within the CRECs is needed to monitor the outbreaks, control the infections, and develop better strategies to fight the bacteria.

Several lines of evidence have shown that high-risk bacterial clones were resulted from the global spread of antimicrobial resistance genes in ECC. It has been reported that the extended-spectrum beta-lactamase (ESBL)-producing ECC belonged to ST66, ST78, ST108, and ST114; ST78, and ST114 clusters, and they carried a resistant gene encoding the CTX-M-15 *β*-lactamase [8]. Peirano et al. reported that ST93 cluster spread in Australia, Belgium, China, Romania, Spain, Thailand, United States, and Vietnam could express a various of carbapenemases encoded by *bla*_IMP-8_, *bla*_IMP-14_, *bla*_VIM-1_, *bla*_NDM-1_, *bla*_KPC-2_, and *bla*_OXA-48_ genes [9]. Another study also highlighted the important role of ST182 in the dissemination of blaNDM-4 [10]. These studies demonstrated that ECC dissemination exists with geographical and seasonal variation in specific regions. However, which carbapenemase genes in ECC clones are passed on in the Heilongjiang Province is unknown. In this study, we isolated 6 CREC strains from patients with infectious diseases in our local hospital and carried out a molecular characterization.

## Methods

### Collection and identification of bacterial isolates

Six CRECs were collected from a total 169 of clinical ECC isolates found from the patients with infection diseases in the First Affiliated Hospital of Jiamusi University during the study period from September 2016 to March 2018. The minimum inhibitory concentration (MIC) of antibiotic assay was carried out with the Vitek 2 Compact system (BioMerieux, France). MICs of Meropenem (MEM), colistin (COL) and tigecycline (TGC) were determined by the broth microdilution method, and results were interpreted according to the Clinical and Laboratory Standards Institute (CLSI) M100-S25 interpretive criteria (CLSI, 2015). The identities of the six isolates were further confirmed by sequencing *16S rRNA* as described previously [11]. The study protocol was approved by the Ethics Committee of Jiamusi University Clinical Medical College for research (20180326).

### Detection of *β-*lactamase genes

The modified Hodge test (MHT) and modified carbapenem inactivation test (mCIM) were performed according to CLSI (2015) guidelines to identify the phenotypes of the bacterial strains. Bacterial DNA was extracted using the boiling method. The ß-lactamases type A encoding genes (*bla*_*TEM*_*, bla*_*SHV*_*, bla*_*CTX-M-15*_*, bla*_*CTX-M-,*_ *bla*_*kpc9*_*),* Type B encoding genes (*bla*_NDM − 1_, *bla*_IMP-4_, and *bla*_VIM_), Type D encoding genes (*bla*_OXA-23_, *bla*_OXA-24_, *bla*_OXA-51_, *bla*_OXA − 48_ and *bla*_OXA-58_), and AmpC enzyme encoding genes *(bla*_ACC_, *bla*_DHA_, *bla*_CMY_) were detected by polymerase chain reaction (PCR) assays, followed by sequencing PCR product as described previously [12].

### Identification of the drug resistant determinants in the quinolone resistance determining region

The determinants in the plasmid-mediated quinolone resistance (PMQR) (*qnrA*, *qnrB*, *qnrS*, *aac (6)-Ib*, and *qepA*) and the quinolone resistance determining region (QRDR) (*gyrA* and *parC*) were examined by PCR as described previously [13, 14].

### Multilocus sequence typing (MLST) and pulsed-field gel electrophoresis (PFGE)

Multilocus sequence types (MLST) of seven housekeeping genes (*dnaA*, *fusA*, *gyrB*, *leuS*, *pyrG*, *rplB*, and *rpoB*) were conducted to investigate the genetic relationships of CREC isolates. Briefly, PCR products were sequenced as described [12], and the DNA sequences were aligned with those in the MLST database (http://pubmlst.org/ecloacae) to identify allelic numbers and sequence types (ST). Molecular phylogenetic analyses were performed by using the Maximum Likelihood method and evolutionary analyses in MEGA7 software. CREC isolates were further investigated by the PFGE. Briefly, extracted bacterial DNAs were digested with *Xba*I restriction enzyme, and the digested DNA fragments were then electrophoresed on agarose gel as previously described [15]. The results were interpreted according to international criteria and allocated into clusters using a cut-off value of 80% genetic similarity [15].

### Plasmid analysis

Plasmid DNA was extracted from the CREC isolates with QIAamp DNA Mini prep Kit (Qiagen, Hilden, Germany) and sequenced [12]. A 10-kb fragment library was prepared with PacBio System (Pacific Biosciences, Menlo Park, CA, USA) and assembled using canu software (https://canu.readthedocs.io/en/latest/quickstart.html). The genomic sequences were annotated using Glimmer 3.02 software. The expression of rRNA and tRNA was predicted using Barrnap 0.4.2 and tRNAscan-SE (ver 1.3.1) software, respectively. The annotated information for the predicted genes was obtained using BLAST (NCBI) and alignment with NR, Gene string, and GO Databases. (Additional file 1).

## Results

### Clinical data assessments

The clinical characteristics of the six patients with CREC infections are summarized in Table 1. The bacterial strains were isolated from the patients in the intensive care unit (ICU), the emergency room, and the infectious disease ward. Two strains were recovered from diabetic patients and other strains were recovered from the patients with fractures, cirrhotic livers, urinary tract infections, and cerebrovascular attacks. The patient carrying strain ECLC6 was a teenager with diabetic ketoacidosis, who had been transferred to another hospital. Most of the patients had increased risk factors for infection or colonization with ECC. All patients were treated with antimicrobials including cefoperazone-tazobactam, aztreonam, and levofloxacin, and etimicin. Out of 6, 5 patients recovered were discharged after successful treatments, one died from bacterial infection.

### Species and antibiotic susceptibility

**Fig. 1:**
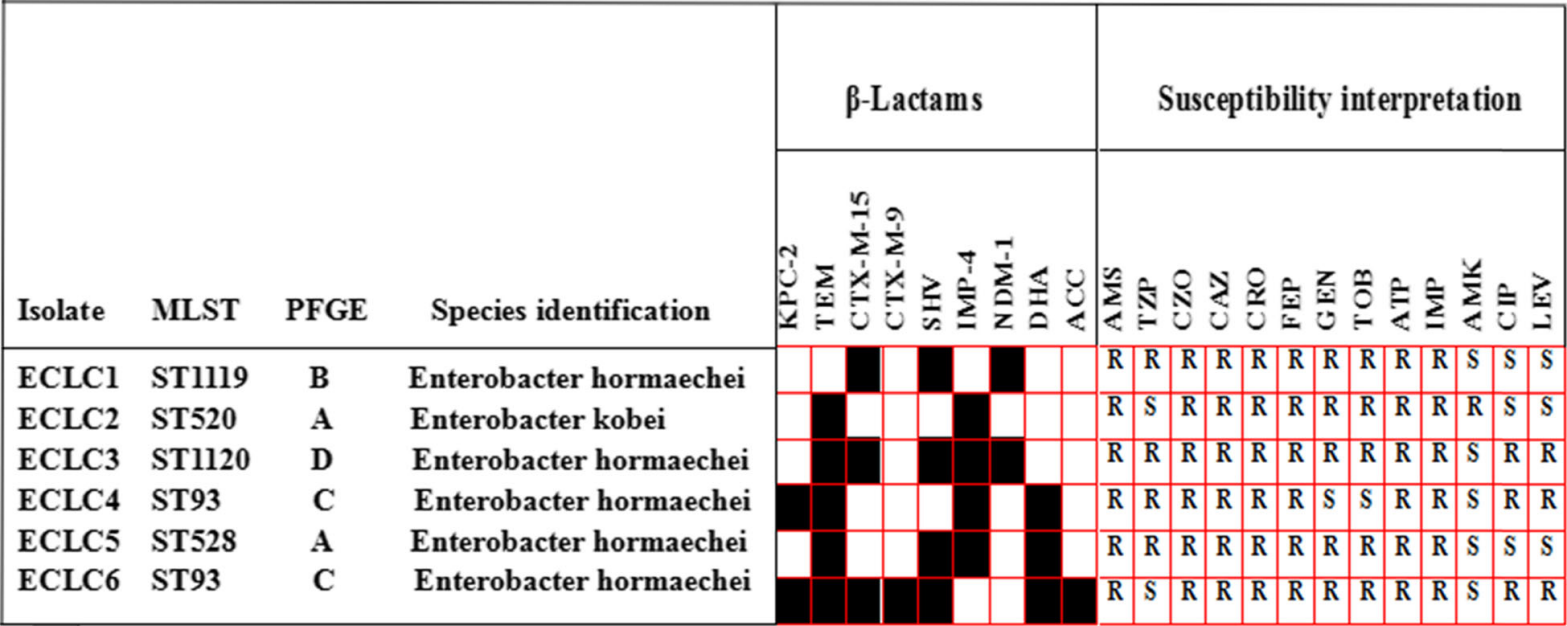
Genetic analyses of drug-resistance determinants in *Enterobacter cloacae* complex isolates. Drug-resistant gene presence was labeled as black shading and absence was presented as white shading for each isolate. The corresponding antimicrobial susceptibility was profiled with the VITEK2 automated system; PMICs for levofloxacin (LVX), meropenem (MEM), colistin (COL), and tigecycline (TGC) were determined using the broth microdilution method. ST: sequence type determined with the MLST. PFGE: pulsed-field gel electrophoresis showed four (A, B, C, D) clusters. R: resistance. S: sensitive

All six isolates were not susceptible to ampicillin-sulbactam, cefazolin, ceftazidime, ceftriaxone, cefepime, meropenem, or imipenem. However, some isolates were susceptible to amikacin, tigecycline, and levofloxacin, and only strain ECLC4 was susceptible to gentamicin and tobramycin (Fig. 1). Because three isolates were resistant to colistin (MIC = 4), we examined whether resistant clones carry the *mcr-1* gene. We performed PCR with primers specific to the *mcr-1* as described previously [16]. We failed to detect the presence of the *mcr-1* gene in 6 isolates.

### *β*-Lactam and fluoroquinolone resistance determinants

Because the resistance to fluoroquinolone (FQ) and β-lactam has been increasing, we examined if these strains bear FQ resistance genes by PCR assays. We found that all six isolates had multiple resistance genes including *bla*_NDM − 1_, *bla*_IMP-4_, *bla*_TEM_, *bla*_SHV_, *bla*_CTX-M-15_, *bla*_CTX-M-9_, *bl*a_KPC,_ *bla*_ACC,_ and *bla*_DHA_. Strains ECLC1-ECLC5 but not strain ECLC6 carried a metallo-*β*-lactamase. Interestingly, strain ECLC3 possessed both *bla*_NDM-1_ and *bla*_IMP-4_. All six strains neither harbor *bla*_OXA − 48_, *bla*_OXA-23_, *bla*_OXA-24_, *bla*_OXA-51_, and *bla*_OXA-58_ genes that encode class D carbapenemases, nor carry *bla*_CMY_ gene. We next examined PMQR determinants *(qnrA*, *qnrB*, *qnrS*, *aac (6)-Ib*, and *qepA)* as well as QRDR genes (*gyrA* and *parC)* by PCR assays. We found that all clones harbored *qnrB* and *qnrS* but not *qnrA*. All 6 strains contained *aac (6′)-Ib-cr* but not *qepA*. Subsequent DNA sequence analyses revealed that the levofloxacin and ciprofloxacin-resistant strains carried gyrAS83F, gyrAS87V, and parCS80I mutations in *gyrA*83, *gyrA*87, and *parC*80 genes in the QRDR (Fig. 1).

### Epidemiological links in CREC

To identify the possible phylogenetic relationships of the CREC strains, we performed molecular characterization and genotyping of the drug-resistant bacterial strains. By MLST analyses, we found that the six isolates could be clustered into five STs. The phylogenetic tree built from the sequence data of the seven MLST genes showed 4 phylogenies of the isolates including ST93, ST520, ST1119 and ST1120 (data not shown). Of them, ST1119 and ST1120 appeared new. It should be noticed that CREC3, CREC4 and CREC6 isolated different departments of the hospital were highly similar. By PFGE analyses, the isolates were grouped into clusters A (isolate numbers 2 and 5), B (isolate number 1), C (isolate numbers 3 and 6), and D (isolate number 4) with a cutoff of 80% genetic similarity. Consistent with MLST results, the bacterial strains isolated from different departments (isolate numbers 3, 4, and 6) were highly similar in their PFGE band patterns.

### Genetic characterization of NDM-1 and IMP-4 in ST1120

**Fig. 2:**
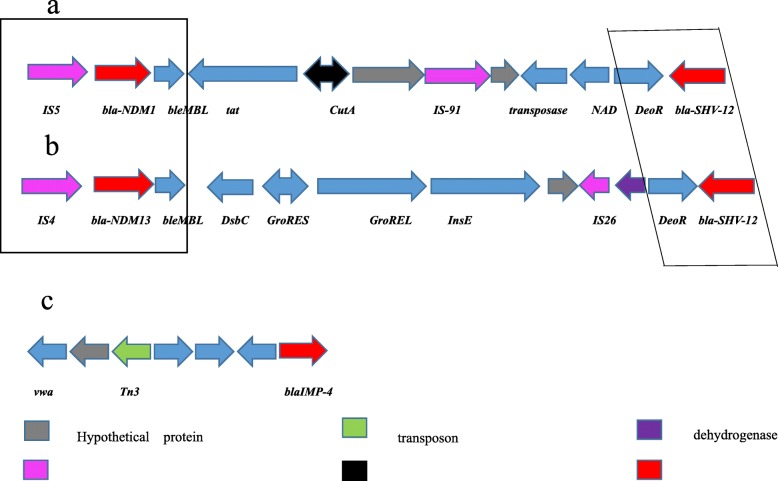
The genetic characterization of *bla*_NDM-1_- and *bla*_IMP-4_- plasmids in ST1120. The *bla*_NDM-1_ gene was in a plasmid containing *bla*_NDM-1_, *bla*_SHV-12_, and *bla*_SHV-35_ resistance genes. The *bla*_NDM-1_- containing plasmid (**a**) was compared with previous published plasmid PZHDC33 carrying *bla*_NDM-1_ (**b**). A partial insertion sequence element, IS*5*, was located upstream of the *bla*_NDM-1_, *ble*_MBL_, and *tat* genes. The *bla*_SHV-12_ was downstream of the *DeoR* gene. The genetic characterization of *blaIMP-*carrying plasmid (**c**) was partially sequenced and presented

**Fig. 3:**
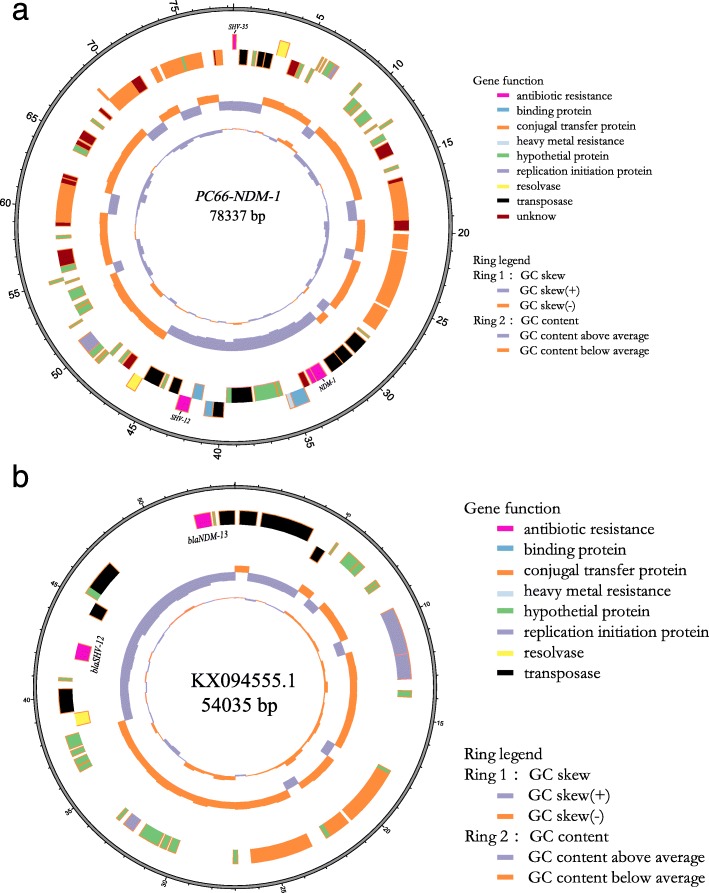
Schematic maps of blaNDM–carrying plasmids. A schematic mapping of plasmid pC66-NDM-1 (**a**). The first ring indicates the coordinates of the complete plasmid circle. The 2outer rings represent the forward and reverse open reading frames, respectively. A similar blaNDM–carrying plasmid of KX094555.1(**b**) was also presented

To characterize the genetic environment of the resistance genes, we isolated the plasmids and sequenced the DNA. Our sequence analyses revealed that the *bla*_NDM-1_ gene was in a plasmid also containing *bla*_NDM-1_, *bla*_SHV-12_ and *bla*_SHV-35_ resistance genes (Fig. 2a). A partial insertion sequence element, IS*5*, was located upstream of the *bla*_NDM-1_, *ble*_MBL_, and *tat* genes. The *bla*_SHV-12_ was downstream of the *DeoR* gene (Fig. 2a). The characterized structure of *bla*_NDM-1_- carrying plasmid is like the *bla*_NDM-13_ containing plasmid, PZHDC33 as indicated in Figs. 2a & b. An insertion sequence element, IS*4*, was located upstream of the *bla*_NDM-14_ and *ble*_MBL_ genes in PZHDC33 plasmid. The *bla*_SHV-12_ and *DeoR* genes were localized at the same positions as PC66-NDM-1 plasmid identified in ST1120. Interestingly, the *Bla*_IMP-4_ gene was localized in a separate plasmid. A detailed map of the plasmid harboring *bla*_IMP-4_ in ST1120 strain was not constructed yet because of short-read sequencing and insufficient DNA sequences. Thus, it is impossible to compare the sequence of *blaIMP-carrying plasmid* with previously characterized plasmids yet. It was likely that the Tn*3* contributed to the transport of *bla*_IMP-4_ (Fig. 2c). The plasmid from isolate ST1120, designated as pC66-NDM-1, formed a 75 kb circular construct which is highly homologous to 54 kb plasmid, KX094555.1 as shown in Figs. 3a &b. The pC66-NDM-1 harbored three antimicrobial drug resistance genes (bla_SHV-35_, bla_NDM-1_, and bla_SHV-12_) in a region of approximately 40 kb from nucleotide position 271 to 42,784, and a heavy metal resistance locus between nucleotide positions 35,333–35,004 encoding the periplasmic divalent cation tolerance protein CutA.

## Discussion

In China, the first CREC was a KPC-producing strain isolated from Shanghai in 2010 [17]. Subsequently, *bla*_NDM_, *bla*_IMP_, and *bla*_VIM_ of ECC were identified from various geographical regions in China as well as in other countries [18, 19]. It has been reported that strain *Bla*_NDM-1_ was the predominant drug-resistant pathogen of CPE in both animals and humans in China and that the bacteria was spread either through tourist traveling or horizontal transmission of plasmid DNA from one bacterial strain to others [19, 20]. Consistent with the previous reports, we demonstrated in this study that two out of six CREC isolates (33%) carried *bla*_NDM-1_. The ST1120 was highly homologous to high-risk ECC clone of ST93 isolated from the Ningxia Province of Western China, where the risk of bacterial transmissions was low because of low population and few tourist travels as compared to the primary global endemic regions of the eastern China [21]. Our studies found that patients carrying the *bla*_NDM_ gene have not traveled to any epidemic areas before becoming sick, suggesting these isolates might acquire and share the carbapenem resistance genes via horizontal transfer between bacterial species.

We also revealed a new CREC strain co-produced both *bla*_NDM-1_ and *bla*_IMP-4_ from two separated plasmids isolated from ST1120 strain from the patient with a urinary tract infection in the Northeast China. Because the CREC isolate was resistant to most current antibiotics and was resistant to colistin too (MIC = 4), we assumed that the bacterial might carry the mobilized colistin resistance-1 (*mcr-1*) gene that can horizontally transfer between different strains of a bacterial species. Thus, we performed PCR with primers specific to *mcr-1*. In contrast to our prediction, we failed to detect the presence of the *mcr-1* in the isolate and therefore excluded the possibility. Our studies indicate that the molecular mechanism of colistin-resistance of this strain is independent on *mcr-1* gene and the genes that confer colistin-resistance remain to be identified.

In the present study, we found both the *bla*_CTX-M-3_ gene and the *bla*_TEM-1_ gene were co-present with *qnrS* genes in a single plasmid in the ST1120 strain (data not shown). This plasmid bearing *bla*_CTX-M-3_, *bla*_TEM-1_ and *qnrS* genes appeared independent on the hosts and was able to transform into a variety of organisms [22], resulting in high-level resistance to quinolones and other antibiotics such as tetracycline and florfenicol. Our studies indicate that the quinolone resistance was not resultant from overexpression of bacterial efflux pumps since the *qepA* gene was not present in the CREC strains.

Mutations of genes encoding DNA gyrase (*gyrA*, *gyrB, gyC, parC. And parE*) and topoisomerase IV (*parC* and *parE*) on bacterial chromosomes were also associated with FQ resistance [23, 24]. In our study, we found existence of mutations in three loci of ECLC5 strain and two loci of ECLC6 strain. Our broth microdilution assay revealed that the levofloxacin MIC for these two isolates was 16 μg/mL and 32 μg/mL, respectively, suggesting that the genetic mutations at these loci contributed to high levels of resistance to levofloxacin.

ST93 of CREC has been spread in other 11 cities but has not been reported in Heilongjiang Province of China before [19]. In this study, we were not only able to detect two ST93 strains present in Heilongjiang region but also uncovered the new strain of ST1120 that is distinct from ST93 in two housekeeping genes (*gyrB* and *pyrG*), emphasizing that more epidemiological tests are needed to prevent the spread of the high-risk clones, ST93 and novel clone, ST1120 in China.

## Conclusions

In conclusion, we isolated 6 CREC strains from patients with infectious diseases and carried out a molecular characterization. We found all 6 isolates harbored multiple resistance genes. Five of them carried *metallo-β-lactamases* and one strain had *bla*_KPC-2_. The levofloxacin and ciprofloxacin-resistant strains had substitutions of *gyrA*83, *gyrA*87, and *parC*80 in the QRDR. Our molecular genetic analyses revealed that 6 isolates belonged to five sequence types (ST520, ST528, ST1119, ST1120, and ST93). We also discovered a new strain of ST1120 that carried two plasmids encoding *bla*_NDM-1_ and *bla*_IMP-4_. Our study has some limitations. First, the study involves a small number of isolates analyzed at a single medical center. Second, although we have characterized the plasmid containing *bla*_NDM-1_, a detailed map of the plasmid harboring *bla*_IMP-4_ in ST1120 strain was not constructed yet because of short-read sequencing and insufficient DNA sequences [25]. Thus, it is impossible to compare the sequence of *bla*_IMP-4_-containing plasmid with previously characterized plasmids yet. Third, we did not examine the colistin-resistant isolates for the presence of other mcr alleles (2 to 9) and chromosomal mutations mediating colistin-resistance in our future studies. The molecular mechanisms by which the *mcr-1*-negative isolates were resistant to colistin but remained susceptible to amikacin and tigecycline remain unanswered yet. Fourth, we did not sequence and analyze the all 6 isolates, and the other 5 strains remain analyzed in future.

## Supplementary information

**Additional file 1.** The plasmid sequence with NDM

## Data Availability

All data generated or analysed during this study are included in this published article and its supplementary information files.

## Abbreviations

CLSI: Clinical and Laboratory Standards Institute
COL: Colistin
CPE: Carbapenemase- producing Enterobacteriaceae
CRE: Carbapenem-resistant Enterobacteriaceae
CREC: Carbapenem-resistant *E. cloacae* complex
ECC: *Enterobacter cloacae* complex
ESBL: Extended-spectrum beta-lactamase
FQ: Fluoroquinolone
ICU: Intensive care unit
mCIM: Modified carbapenem inactivation test
MEM: Meropenem
MHT: Modified Hodge test
MLST: Multilocus sequence typing
MLST: Multilocus sequence typing
PCR: Polymerase chain reaction
PFGE: Pulsed-field gel electrophoresis
PMQR: Plasmid-mediated quinolone resistance
QRDR: Quinolone resistance determining region
ST: Sequence types
TGC: Tigecycline
