# Supplementary material for: Molecular characterization of metallo-β-lactamase- producing carbapenem-resistant Enterobacter cloacae complex isolated in Heilongjiang Province of China
Source: BMC Infect Dis. 2020 Jan 31;20:94. doi: 10.1186/s12879-020-4768-7 (PMC6995058; doi:10.1186/s12879-020-4768-7)
Supplement: Supplementary file 1 — Additional file 1. The plasmid sequence with NDM [file 12879_2020_4768_MOESM1_ESM.docx]

The plasmid sequence with NDM:

TTTTCGCTCCAGCTGTTCGTCACCGGCATCCACCCGCGCCAGCACTGCGCCGCAGAGCCACTACTTTAAAGGTGCTCATCATGGGAAAGCGTTCATCGGCGCGCCAGGCGGTCAGCGTGCGGCCGCTGGCCAGATCCATTTCTATCATGCCTACGCGGCCCGACAGCTGGCTTTCGCTTTGTTTAATTTGCTCAAGCGGCTGCGGGCTGGCGTGTACCGCCAGCGGCAGGGTGGCTAACAGGGAGATAATACACAGGCGAATATAACGCATAACCACAATACATCCTTGAGTGAAGGCCGATAAAGGCGAGTAAAGAAGCGACAAATAAGAATAACCCGGCGTTGGCACTGTTGCAAATAGTCGGTGGTGATAAACTTATCATCCCCTTTTGCTGATGGAGCTGCACATGAACCCATTCAAAGGCCGGCATTTTCAGCGTGACACATCATTCTGTGGGCCGTACGCTGGTACTGCAAATACGGCATCAGTTACCGTGAGCTGCAGGAGATGCTGGCTGAACGCGGAGTGAATGTCGATCACTCCACGATTTACCGCTGGGTTCAGCGTTATGCGCCTGAAATGGAAAAACGGCTGCGCTGGTACTGGCGTAACCCTTCCGATCTTTGCCCGTGGCACATGGATGAAACCTACGTGAAGGTCAATGGCCGCTGGGCGTATCTGTACCGGGCCGTCGACAGCCGGGGCCGCACTGTCGATTTTTATCTCTCCTCCCGTCGTAACAGCAAAGCTGCATACCGGTTTCTGGGTAAAATCCTCAACAACGTGAAGAAGTGGCAGATCCCGCGATTCATCAACACGGATAAAGCGCCCGCCTATGGTCGCGCGCTTGCTCTGCTCAAACGCGAAGGCCGGTGCCCGTCTGACGTTGAACACCGACAGATTAAGTACCGGAACAACGTGATTGAATGCGATCATGGCAAACTGAAACGGATAATCGGCGCCACGCTGGGATTTAAATCCATGAAGACGGCTTACGCCACCATCAAAGGTATTGAGGTGATGCGTGCACTACGCAAAGGCCAGGCCTCAGCATTTTATTATGGTGATCCCCTGGGCGAAATGCGCCTGGTAAGCAGAGTTTTTGAAATGTAAGGCCTTTGAATAAGACAAAAGGCTGCCTCATCGCTAACTTTGCAACAGTGCCACAATGTGCGTAATGACCCCCCTGACCTCCAACCCGTCCTCATCGTCATCAGGTATAGCCCTGAAGTTATCCAGCTTATCGAGTTCCTGGAGACACCGCTTGGGGTGTAGTCGCAATCGCATGATCCTGAATTCTTGTTCCAGACACAGAGCACCAAAGACCCGTCAACGGGCGTGCCCCCAAAATCTACTATGAGCAGCGCACCAGGCTTTATCCCTTCCCGGAAAGAGTAGGTATCACTTCGAAAAAAGAATACTCCCGGCGCTCCTGTATTACAGTGCTGATCGAGTGACAGCCTGTTTTTCCTGGTAGGTCGGTTGCTGGCGATGGAAAACCCATTTTTAACGGCCTCCGTTGGGGTTAAAAAGCATGAATGTCCGGTTCTCTCCTTCCTGAGTGGAAATGTCTTTAAAAACGGTCACGTAGGTTTCAATCCACTGATTTGCTTGGCGACAACTCCAGTGAAAATTAATCAGCGCAAGCTGGCTGACAAAGGCTTCTGTCGTAACTGTCTGACGACCGTTCGGCTCACGCTTAATGCTCGCACGCCAGGCTGAGTCAATTTCATAGTGTCTAGGCATGAAAAACCTCCGATCAATATAGCTGTATGCATATGGGTCTTCCCTTGTTGTGGTGGCTGAAGGCATGATAATGGTGTATTAATCGCCAGAGGTCACCGCCATGGACGAAAAGTCCCTCTACGCTCATATTCTCAACCTGTCCGATCCGTGGCAGGTAAAGTCCCTTTCTCTCGATGAAAATGCCGGTTCTGTTACTGTCACTATTGAGATCGCTGAAAACACCCGGCTAGCCTGTCCGACCTGCGGTAAATCCTGTTCTGTTCACGATCACCGTCATCGTAAATGGCGCCATCTTGATACCTGCCAGTTCACCACTATTGTTGAAGCCGATGTTCCACGAATTATGTGTCCGGAGCATGGCTGCCTGACGTTGCCTGTTCCGTGGGCTGGCCCCGGAAGCCGGTATACGTTGCTATTCGAATCGTTCGTTCTCTCATGGCTGAAAATCAGCACCGTTGATGCTGTCAGGAAGCAACTTAAGCTCAGTTGGAATGCGGTTGACGGCATTATGACCCGGGCAGTTAAGCGAGGTCTTGCCCGGATAAAAAAGCCATTATCCGCCCGTCATATGAATGTGGATGAGGTCGCCTTTTAAAAAGGACATCGTTACATAACGGTGATCTCCGATCGCGATGGTCGGGCGCTGGCCTTAACGGATGATCGCGGCACAGAGAGTCTTGCCGGCTATCTTCGCACGCTCACTGATGGGCAGTTGCTGGCTATCAAAACGCTCTCAATGGACATGAACGCGGGCTATATAAGAGCAGCGCGTATCCACTTACCCAGTGCGGTTGAGAAAATCGCCTTTGACCGCTTCCATGTGGCGAAGCAACTGGGCGAGGTAGTTGATAAAACCCGTCAGAATGAACATCCGCACCTCCCTGTTGAAAGCCGACACCAGGCAAAAGGAACCCGCTTCCTGTGGCAGTACAGCGATAAGTGGATGACCGAATCCCGGCAGGAAAAGCTGATGTGGCTGCGTGCACAGATGAAGCTGACGAGCCAGTGCTGGGCGCTGAAAGAGCTGGCAAAGGATATCTGGAACAGGCCATGGAGCGAGGAAAGACGGAGTGACTGGCAGAGATGGTTGGCGCTGGCGGCTAACAGTGACGTTCCCATGATGAAAAATGCCGCGAAAACGATAGAAAAAGGCTGTACGGGATCCTGAATGCGATGCGACACAGTGTCTCAAACGGAAATGCGGAGGCACTTAACAGCAAGATCAGGCTGCTGAGGATAAAAGCCAGGGATACCGAAACCGGGAGCGCTTTAAACTGGGGTGATGTTCCACTACGGAAAGCTGAATATGGCGTTCTGAGCCTTCCCACCATGATCGGGAAGACCCATGCATATACAGTATAAAGAATCAGTTTTATTCTTCAACACACTGTTTATTATGTTGTAACTACTCCCTACCTTCAGGTTCATGGTTCATTTTGTTAACGGTCTGTTGCTTCACAGCTTTGCTGATAGTCGCCCGGCTACAACCCAGCACTTTTTGAATCTGGCTCCAGGAGCTGCCGCTCGCAATCAGCCTGTTTATGGCGTCATAGCGAGACTGATTAACCTGGCGGCCTTTATACTTCCCTTCCCTTTTCGCCCTGGCGATCCCCTGCTGCTGCCGTTCCCGGCGCTGTTCATAGTCTCGCCTGGCGACGGCGGCCAGCATATCCAGCAACATATCGTTTATGGCCGAAAACATTCGGCTGTCAAAATCATTGTGACCCGATGCGAGCCAGGTTGTCGGAACGTTGACGGCCACAACCCGGATATCTTTCTGCCGGATCATTTTCTTCAGTGTGTTCCAGTCCTCCCCTACCAGGCGTGAAAGCCTGTCCACATCCTCTACCAGCAAGATATCGTTTTGCTGACAATCTTTCAGGAGCCGGAACAACTCAGGGCGTTCAAGCTTAGAACCTGATTCATTTTCAATGTAGTAGTTACAGATGCTCAGGCCGCGCTCGATGGCGAAGGTATTTATCGTGTCCAGGGCGCGTGTTGCATCCTGTTCAGTCGTGGATGCGCGTAAGTAAGCTCTGACAAAGCCTTTCGGTACTGTTTGAGTCATTATAGGGAACCAGTTCATTTTGAGTTAACCAGACACACCCATACAGTACCCATTTCAGTTGGTTCAGGGAAGGCACACTCAAAAAGAACCAGCAAACAGACCCCAGCCCTATAGTGACAACGTCACACTAAAAATTTACAACATAAAGATGATGTAAAGACACACCAAGATTTACACCATAAAGCGCACATAAAGACACAGCAACATTTACCATGTAAAGACACCATAAAGATAAATATCTTTATTTGTGCTTTACATGGTAAAGCCATTGCATTATAAAGACACTGTAAAGACAAAAGTATCTTTACGAAAACACACCATGTAAAGACATTCATATCTTTACATGAGCTTTACAACATAAAGAGAGGGATTCTGAAATGGGTAAAATCTTGCTTGTCGTATCAGATAAAGGTGGGGTAGGCAAAAGCACCTACGTGGCTAACACAGGCTCAATGCTGGTCAACAAAGGTAAGTCGGTAATTATCCTGAAGACAGATAAAAACCACGATCTGTTGAGCTGGAACGAAAAGCGGACAGATAACGGCTTACTCACTATCCCGGTTCACGCAGCTTACGGAAACGTCAGCAACGAAATTAAGCGCCTTAGTAAGCTGTGTGAAGTTCTCATTGTCGATTGCCCCGGCCACGACAGCCAGGAGTTCCGTAGCGCCCTGACAGTATCAGATATCGTGGTGACACTGGTTAAGCCGTCCTCTGATTTCGAAAGCGAAACACTGACCAGTGTTACAGAGAAAATCCGCACCGCTCAGAAAGCTAACGCAGCTCTGGAACCGTGGGTGCTGTTCACCAGAATCAACTCAGCGAAACCCCGCCACAGAAAAGCGGCCATTGATCTGGATAAATTACTGCGTTCGGACAACATCTGGATTCAACCTCTGAAAACCCGGATATCCGAGCTGGATGTGTATGAAAGCGCCTGTAATGAAGGGGCAGGGTACATGACGTTAGCCGGGCATCGAGCCTTTCAACAGCGAAAGCACAGATTGAACTTGTGGCACAGGAAATTGGCATTTTATAGAAATATCAACAATATTCATCGAAATCTTTACATTGTAAATACACCATAAAGATACTATGATATTTACAATTAATGCATACCGTACAAGGGGAAATTTCATGGCTTTAAAACTAAACCGCCCGAACATCGATGAATCTCAGCAACCAGCAACAAACGCAGAAACCGCGCGTTTTATCTCCGGCGCTACCAGAGCGCCTGTAGCGGGTAAACCAAAGTTGATGAACTTCAGACTGCCAGCGTACTTTGAGGAGATCCTGGAAGCGGAGGCGCAGAGAACAGGCCAGAGCAAGACAACCGTTCTAAAGGCAGCGCTGGCCGCATTCGATAACCTGGACGAGAACCTGAAAAACCACTGGCTGCTTGAATCTGCAAAATTAGGGTAGCGTTATGGCTAAGAAAAAGAGATATGTTGTGATGGTTAGAGATAAACCAATACAGCGGCAATCAGCGATTATTAGCCTGGTTAGTATGGTTCGCTAACAGGCATAATAAGGCCGTTGCCTATGACTGCGGAGAATGGATTGTCGAACCATCACATTGGTTAAGAATTGGAAATCCAAAATAGAAAGAAACCTAAGGAAAGAAAAATGTTCATATTCAGATATATTAAAACTCTTATTAATATCTACTTGTTATACATCCAACCGATATTGGGGATGTTCCTTATAGGTATAGGCTTATGGGGGTTCTATGCACCTGATGAGGGAACTTGTACTAATCTCAATATTGAGTTATGCGCTGTATCAGCTATTGATGTTCTTAAGCCTGCTATTTTTGTATTTTTAGGCATTATTACCGTTGCCGGATGGTACTTTACCGAACTTCAGCCGAAGCTAAGGGGGAGGGCAAGTATATGAAGGTAAAAACAGGTGTAAAACCGGAAGATTTCGTTAAGTAAAAACCGAGCCTAAAGACGTTAACCGTGGGGGATTTATGAATAAATCATGGCTTGAACAACAGGTGCAAAATGAAAAATTAAAAAAAGTACAGGAAGAGAAAAAAGATGGCACTATCCAAAAAGGATTGTACAGGGTTCGTATAAAAGACAAATACAGGTGGGATTTGTGGGAAAATGATTTCAGAGTTGTTATTGCTTATTCGAAAGATGATGCAGAGCTAAAATTTACAGAAATGACAGGTTTTAATGAGCATTACTATATAGAAAGCATTGAAAAATAGTGCTTTCAGATATAGAAAAAATCTTACTGGAAAACTGAAAAGATAGTTATCCAGAGGTTTAGTTAAATAATGGCGTCAGTTTTTCAACCAGTAGCAGATCGCTTAAACCGTCTAAAGAAACAACTTCTATACCAGAATCAACAAGTGAATCAACTCCACATACTTCACCGAAGCCGATAACCATAATCGGCTTTCGGTCTATCAAATCAAAATCACCAAAGGCAATATTACACTGTTAATCGCTTCAAACTCCTGAAAAAACATTTCAGAGCCGTATTCGCTGGCAGGAAAAAGAATCTCACTTAATCTCAAACCATAAAAATATGGTAGTGTTCTTTAAGGTTATCCTTTAAAATATTAACAGCCTTAGAATATTTTTCGTTAATAAACTCTTTAACCTGATATTTCATAACTCTCTCCAACAAGAACCGACTGTAGGTCACCGGGCAAACGTTGCGGAATGGCGTCAGAGACGTCATTTTGCGGCGTTTGCCCTATCCTGCATCGCAGTGAAAAGAACTCGCCTCGTGCGCTATGATTAACCGATGATAAAAGGCTGAAAGAAGCACTGCAAGCCGACTGGATAACATTGTTGCTTCACGGCTGATCATCATTGGTTGCCTCAGTGCTAGTGTGCTTTGCATCAGGAACCAGAAGACGATACCACGGCATTTTTTTGTTAAGCAGCTCCATCAGGGGAGTGGCAAACAAACTCAATCCCCACAGCGTTACGATTACTGGAATCGTGAAATTTTGCGGCGTCCAGAACTGGCGCTCAAGGTGGAACCATGTAACAACACAGAGCATTCCACCAATCCAGAAAATAGCTTTACTGAAAGCCCCCAGGACTGCCAGAGAAATAACAGAGACTAAATGCAGTGCGCCAGCCGTAGCAAGACGTAGATAAAACCAGAACCATTTAGCGTGCCGGATAACCGGGTTACCGTTCGTTTCTCGCAATCGTTTTTTACGTTCCCGGATGATTTCTTTTTTCAGCTTACTTCGTTCGGCTCCCTGCGGAAACTCATATATTTTAGCCATGTTTTGCCTCACTTATTCTTTTTTGGTGGAACGACTTTGTCAAACTCCTCTAAAAATGCCATATCATCTTCCGAAAAGGATGAGGATTTTTCATTTATACTAAACTCAAATCTAAGCATGTGAGCCATTCTCCCTTTTTTCCCAACAACAGAGAAAGATAACTGATTTATTTCCGTTTTCTCAATTATCTCATTAATGCTTTTATTGATAACATCACGAACAAAAAATGAGTATTTAGGATACTTATATTTCTTCTCCCCTTCCTCAATAGTATAAAGTCCCATTTCCTCTTTTAAATCATCCACGGATATATCAAAAGATTTTTTTGAACTATTCTGACTGTAATATTTCCTAATGACTTGATAAAGGTTTGTTGAGTTTACACTGTTCAACAACACAACTGATTTCAATACCTGAGTGGTATATCTATTTTCAGAACCAATCAACTTAGAAAAATATCGCTTAGCTGTATTTGTGAATTTTATCCGTACAGTTGCAAGATGATCGTAATAGGCACAGAATTCTGTTAAGTTTAAATCCAAGCGATCAGGTATTTTATTCTTAGATGATAGTATCCCAAGTTCATCAGCCAAATTTTTTAAATCATCTCTGTTTAAAACTAACGTAGTTGAAGAAAGAACTTTAGCTCCCTCTTTCAGAGCTGCATAAGAGGCATATTTATCTAAAGAAGTAGCTTTTGAAAAAAAGTCAGCATCAACCTCCAAAATATCATCATCTAACATTCCTTTGTATCAATCTGGCAAAGAGCCAGAAAAAGCACTCTTTTTGCTGAAAGGGAAAGGGATGCAAGAGTTACCCCAACTTCATTTCTATGTCTAACTTTCGTTAATCCCGTTACTGTCTTCACGATAAGCCTCATATAACCTTTCCTATGTAGGCAAATCATAACCCCACAAAGGTTGTAATTCAAGACAACAATACAACCTTTAATTAAAAAACCTCTCACCTTTAGATATGAAGATCTGAGGATCTTCATATCTAAAGATAGCAACAACCGCATGTTTCACTTGATGTTCAGTCAAACTGTGGAAACCGTTAAAAATCTTCTCACCTTACGTGAAAAATCCTCTCATCTTTAGTGAAAAATCTAACTACCTTAACTTAAAATCTTCTCACCTTACGTGAAAAATCCTCTCACCTTACGTGAAAAATCCTCTCACCTTTAGCAATTTTTTTCCATAATTTTCAGGTAATTGAAGCGAGCTAAGTTTTTAAGTTTTTAAGATCAAGAAGAAAATATAAGGCTTTTAATGCACAGCGTCTGTGGATAACTCAGCATAAATCCAGAGGATAGATCCGTCTGGCCTCCCTGAGCACTGCACCACCAAAAATTGAGCAAATATTAACCTTAGCGCGTAAGTGCTCTATTGAAACCCCGTGGAAATTCCGCTTCCTCGCTCACTCGCGGGCTTCGCCCTGTCGTTCGGCTGCGGCGAGCGGTGTAATCTCTCTAAAAGATCCAGACGGGATCCTTTTCCGATCCAATTCTTAAATGCTCTCAAAAGCCCTCAGAGGCGTTTTAACCGCTTTAACCCGGTCATGGTTCAACGGAGCGGGTAAAATGCGCTTAGAACGTCATACAGCTCGTTTTTAACCTGATCCCTAATCAGGGGGTATAACAGAGGCGGCCAAAAAATCCGGCCTTGCTCGTCAGAGTATCTACCGCGCATTATCCCCGAACGGAAACCCGACCATCACAACACTTGCACAGCTTGCAGCAGTAGCCGGGTTACAGTTCACTCTCAGTAAGCCCAACCACTAACCTTTCAGACAAGCCCCTCATACCGGGGGATTATTTCTTATGCCAGCAAAATTTCACGACCGGCGAGGATGGCCAAGTGTTTTTTGCTGGCAATTTTCGTGCAGCAGCTGGCGCCCGGATTCATTTTTTGCTGTCAGGCATCAATCGGTTTCAGCAACCCGCCCCGTAATTCGCTGCTAACGCATCTCATAACGCGCAGATACAGCGCCCCGCGCCAGTGTTTCACACTGACCCGGCGTACCGTTCGCAAGCTCACGCGCTAACTGCGACAGGCTAAACATCGTCTGATAGTCAGAAATTGGCCTGGCAGGGGTGAGGGGGTTATCGCTCTCATTCGGGCTTGTTGGTTCGCCAGTCCTGGCGAACGTCTCCGGCGCGATGTTACGACAGCGCATTATGTAACTACAGTTCGCGCCTCTTTCGGAAATCCCTTCATGCGCGGTAAAGCAATATACATCTTTATGTAATCTTTACAATGTAAAGCATTAAAAATGATTCCATAACCATCATTTGATGGTATAATGAATTTGTTGGTTGGGAATGGTTCCCTGCCAGATTCCAGAAGCCCACACGGGCGGGAGCGAAAAATGAATATTCAGGAAGCATTAAACGTTTTTGGTTTATCCGGTGATCTGACTGAAAAGGACATCAAGGCGGCATACAAAAAAGCGGCTTTAAAATATCATCCAGACCGCAATCCATTGGGTGCCGAGCTGATGAAAGCAGTAAATGCTGCGTTTGATTTTCTCATGGCTAACATTGATAAAATCAATCAGTTTCAAAGCACTGATGAAAACGCACGTTACAACTACGGTGAAGATCTGGAAAAAGTTCTGAATACGCTTTCCGGCCTTACAGGGATTGTCTATGAAGTTATTGGTAATTGGGTCTGGATTAGCGGAGAAACTAAAGAACACAAAGACATTTTAAAGGAAATGGGCTGTAAGTGGGCATCTAAGAAAAAACAATGGTTTTACCGTCCTGAAGAACACAAAAGCCGCTGGAACCGTAAAGAACACAGCATTGAAGAAATTCGTGAAATGTATGGTACAGCGGGTAAACGTAAGGCGTCAGGCTGGACACGTGTAGAAGCAAGCGCATAACCGGAATGGGGGCGAAAGCCCCCTAGAACATGGCAAAGATTGATTCGGAGACACGTTGATGAAAAACAAGACCATAACGGAAGCGGAGCTGATAAACATTTTTGAAAGTTACGGAGCCTACATTTGCCCGGATGAGATCGAAGTTACAGCCAAAGAATGCAATGAAAATGGTTCAGTCTTGCACCGTGGCTTAAACGCAGAAGGATGGGCGCATCTTTTTGCGAAGGAAGAAGCATATCAGCAGGAATGCGAAGCCCAGGAAGCTGCAAGTGATGATGGACACTTTGATGAATAGTCTTAAAACTGCCGTAATTCGATTTACGGCTTTTTTATTGAGTTCAAAAGGCTGAATCGATGAAAGAACAGAAAAATTTCTTTGAACGGTATAAACCGGTGTTTGAAATCGTTTGTCGAATCCTGGGGAACGGCTGGCGTGTAAATCTGCTCGATGATTGCCAGTACAGGATTAAGCTAACCTCACCTGATTTTAAAAATTACTCTATACATATTCGAATGGAGAAAGGAAGGTTAGTTATCATTGGAAGCGTAGATAGTCGCAGCTGGAGAAGTCCTTACCATACTTGCACAGTATCGCCAGAGCGTAACCCTGTAGAAATCGCCGCCGATATTGAAAAGAAAATCCTGTCGGATGCTTTAGATAATGTAGATATGGCGCGAGAGTATGAACAACAGTTACAGCAAAAACGCGAGAAAAAACTGATCCTGAAAGGAATGCTATCTCGTTTAGTGCATCTTGAAAGCTGGCATGGGACATTGACCGGGTTTAAAGTTGAGAATGGGTTAGATGGTAACGTATCAGAACGGGGCGACGGATATGAAATGGTAATTCGTGGCCTGAGTGTAGATCAGCTCATAAAGGTAGCGGGGTTTATTAAACAGCTATGAGGCAAAAAATGAATGAAGTCCTGAGCGACAGGAAAAACAAAGGTAATGTAACATACAGGATTGTTGTGTCTGAAGATGCAACACGTTTGTTTATTGAACTTGAAAAACTGATGAAGGTACGCTCTAAAGAAGCGGATAAAAAAACAACTAAAAAGATTGTTTTCCAAAAATCATTTTATTACGAAGAATTCTCGAACGTTAAAGATGAAATTGATGATCCAATTTTATTGAAATTCTATACAGATACTATTGTGAAGGTACAAAATCATGAGTTTTAAGCCAACACCTGAAGAGGTGAAACAAGCACGTATTAAAGCTGGCTTTACGCAGCAGGAAGCCGCTGAACGGTTTGGTTTTACGTTATCAGCCTGGCAAGCCAAAGAAACATCCGGTAAAACGTCTCGTGGTTTAGCCGCTGGTACGTATGAATTACTTTTGCTCCTGGCTGATGAACATCCAGATTACCAGCTCGTTAAGAGAGAAAAAAATCAGGATAAAGTGACTAAATAAATTGCATTACCATCATATGATGGTATAATTTATTTGTAGGTCGGGAATAGTTCCTTACCATGATCCAGTAGCCCAAACGGGCGGGAGACTAAAATGAACACGTATGTTATCACTTTCCAGGCTTTAAACACTAAGAAAGAAAAAATCACCTGCTCTGCTTTTGTCCATTCAGAAACTCTCTTCCTTGCCGTTCATTCTTTCGAAGATAAAAATCGCGGTCGTGGTTATGTTATCACGTCTGTTAGTGAATTGTTGTCCGAGGAACTGACGGCGAAAAATTCCCTCAAGAAAAATATTAACTTTTGGTTTAATGAGTGTGGTTTAAGTAAATCTGAGGTTATAAACGAAGTAATTAACTGGAAGAATTTCGCATATACCCTTAAAGAACTGGAGGAAGCCAAAAATGAAGCGATAAGAGAACTGCGGAGTTAATCAATTAGGGGTGGTATCAGGTTGTGGAGGCAACCTGATACCGTGACACTCAATTCCCTCGCGAGGAATATAAAATGTCGGAACGCATGTTATCAGCAATACAGACTGTTGAAAAGGGTGGGCGTCCGGTTTTTCCCTTGATGCCATTCTCTGCTTTTCCTGAGTACATGGCATTACTCAGAAAAGCCCTGGAAAAGAAAGAAACAAAAGCACTGATAGAAAAACAGGAGGTGCTATGAAAAAACAGGAGTTTTTAGATTTTATCAGTGCAGAACAGAGGCGGGGAGCTGTCCGGTTTTCGCTGGGATTTAACAGCAAAGGGGAAATTGTATTGCACTGGACTAACGAGGCCGGGTTAAGAGTCTGGAGTATACTAAGCGGTAACAGGGGAAAAAGTCCCAGCCGGGCAAACCGGGAAAGAATGAGTAACCTCCGCCGCTGGCTCCATGATGCCCGGCAGGGCATGGAAGGCGACACACCAGAGGCAGAATAAAATTCCCGTTCCTGAATCGCCGAAGCCCTCCCGTTTACGGGAGGTGCTTTTTTTGTATATTCCGTAAAACAGCGCTCGCGCCCCCTGCCAGGCCATTCGCGTAGCGAATTTATTTCCGCCCAAGTTAGCCGCGAATCCGTTGTCTCCGGTCGCAGACGCACCAGATCACAGCCACGATAGCCTTTCATTTTTTGCATTTCGATGGACGCTGAAGCACAATTTATGTGTGGAGGTAGTTCACTGCGATGCAGGATAGGGCAAACGCCGCAAAATGACGTCTCTGACGCCATTCCGCAACGTTTGCCCGGTGACCTACAGTCGGTTCTTGTTGGAGAGTTTTATGAAAAAAGTTCAATTCAGGATTGAAGAAAGCCAACATGATGATCTACTTGATTGTCTTAAAACACTTTATCCAGATGAACCGGGTTTAACCGTGGCAAAAGGAATGAAGCTGCTTGCCAATGCTTTACTGAAAAGCAAGGTTAAAAATGAAGATGTTAATGTGTCAAAAGACAATGATGATTTTATCAAAACCACAATGTATTTGACAGGGAAACAAAGAACTTTAATAGAGAAAGCGGCTCATCGTCATGGCTGGAATTTATCCAGAGAATGTCGTTATCGAATCCAGACGACACTTGAAAATGAACTTGATTTTTTCGATCAGGAACTTCTGGTGATGAATCGTTGCCGTAATGCAATAGATAAAATAGGGCGAAACTTTCATTATATTATCGTTAATGATAATGCCAGAGTACTTGATAAAGATGGGTTCTATCAGGACGCTGAGCGGTTGAGTTCAGAGATAATGAGTCTCAAAAGTGAGTTTGAAAACTACATTATGTTATGCAAAGGGCGAACTGTTTCTAACAAGGTTGAGGTGTAATCATGGGCGTTTATGTTGACAAGGAATTCAGGGTTAAGCGTAAGTCCTCTGAAGCTGGCCGCAAGTCTGCTTTCGCCCATAAAGTTAAAAATGGTGGTAAAAACTATCAACGCAACGTTCAGGAACGTATCAACCGCAAGGGTGCCAGTAAGGAGGTTGTTGTCAAAATCTCAGGAGGTGCGATTACCCGCAGGGAGTTCGAAACAGTATTGACTATATGAGCCGGGAGTCAGAGCTGCCAGTGATGAGTGAAAGCGGTCAGGTATGGAAAGGTGATGAGATTCAGGAGGCTAAAGAGCACATGATAGATCGTGCTAATGACCCTCAAAATGTTTTCGATGATAAAGGTAAGGAAAATAAAAAAGTAACCCAAAATATTGTGTTTTCACCGCCTGTATCAGCAAAGGTAAAACCTGAAGATCTGCTTGAGTCTGTCAGGAAAACGATGAACAAGAAGTACCCAAATCATCGTTTTGTGCTTGGCTATCATAACGACAAAAAAGAACATCCACACGTGCATGTCGTTTTTCGTATTCGTGATAATGACGGTAAACGAGCTGATATCAGGAAAAAAGATTTACGGGAAATTCGTACAGGTTTTTGTGAAGAGCTGAAGATGAAGGGCTATGACGTTAAAGCCACTCATAAGCAGCAGCATGGACTTAACCAGTCTATTAAAGATGCGCACAAAACAGCGCCAAAACGACAGAAGGGCGTTTATGAGGTTGTTGATGTTGGCTACGACCATTATCAGAACGATAAAACCAAACCAAAGCAGCATTTCATAAAGCTTAAGACACTGAACAAGGGCGTGGAGAAAACATATTGGGGGGCTGATTTTGGTGAGTTAACCACTCGCGAGAACGTTAAGAAAGGTGATCTTGTTAAGCTGAAGAAGTTAGGACAAAAGGAGGTGAAAATCCCTGCACTTGATAAGAACGGTGTACAGCATGGCTGGAAGACTGCACACAGGAATGAGTGGCAGCTAGAGAACCTGGGGGTTAAGGGCATAGACAGAATCTCTTCATCCAGTAAAGAGCTGGTGCTGAACAGCGCGGAGATGATTAAGAAACAGCAACTCCAGATGAGAAATTTCTCGCAAATAAAACAGTCGATGATTCAAAGCGAACAGAAAGTGAAAATCGGAATTCGTTTAGGATAATTCATACTCTCCGGTAACTTATTGATTAAGATCGATTTATTTATCTTGTTAGCAGAGTTTTTGTAAAAAGTAACTAGCGGTAAAAAATTTAGTTAACTTGACTATAAAATAAGCGCATGGTGATCAATCGATCTGCTTTTTTGTTATCAAATAGCATATGTATCTAGATATAAATAAAATACAAGCACAAACAAAATGAAATGTGTCGTAAATGCGATCTAAAGATCTGCTTGTGTGGTTGAACGCAGCACATGTAGCTGATAAAGTTTTTATGAGATGTAAATTTGTCCTTTTGTTTTTTTTCTCTGATTCGGGGTCATCCGAGTCGAGAGAGCGCCTGAAAGGAAAAACTATGGAATGGTATGTTTTGCAATTCACCACTACGAGATTTGCAGCAGTTTTTGCTCATCTCGAACGCCTGAACTTCTCTTACTTCTGTCCTATGGAGACTGAAAGGTATCGTCGCCCGGATAAGATAATTTCATATCGAGAAAGACGTTTGCCGCTTTTTCCCGGTTATCTTTTTATCCAGGCAGATTTTGAAGAGGTTCATTCCACTACAATAACTGCTATCCCTTACGTACAACGCTTTATCTCATTTGGCGGTGAGCCTTTACCTGTACCAGAAGATGTGATGGCGGAGCTTTTGTACAGACAATCACATACAACAGCTCAGGCAAATCTTTTAAGGAAATCAATTCCACACGATTTTGCTGAAATTCTACTCATGGATAATCCGCAGCAAAGAAGTATGGCTTTCATCCACTACATCACAGAGAGAAGCTTAACCCACAAAATGAAACGGAAGAAAAATGACTGCTACCCGAAAAAAAACCGCAACGAGACGCAAGCCCCAACGTAATACACTCTACCTGCCCTCAGCTTCTCGTGAGGAACTGGATAAAATTGCTCTGGAGATTGGCTATCGCAGGGGGCGACGTATTTCAAGCTCTGGTGTTGTCCAGTACCTCATAAAAAAATATTCCAGCCAGGCTATTGAGGAGCTGATAAACGAAGACGATGAAGACGATGACGACTTCGATGACTAACCATTCAGTCTAAACTTTCCGCGCTGTTCTGGCCGGGAGAAAATCATGTTATCTACTACTGCTTTTGCGGCGCTTGCTTTGCAATGCGCGGCCAGCGTTCATCCCGATACTGCGCATGAAGTTGCAAGGGTTGAATCTGGTTTTAACCCATATGCTATTGCCGAGATAATACCGAAGGTTGAACGTAAGCCCGGTGACAAAGGCGTAGTGTCCTACTTTCCCAAAACTAAGGAGGCTGCACTCCAGATCGTAAATCAAATTGAATCACGAAATCATCGTTACTCAGTCGGTCTTATGCAAATAACGAGTACGAATTTTGCAAACTTCAATACAACCGCTGAGAAAATGTTTGACCCCTGCGAAAATCTCAAGGTTTCAGAGCAAATTTTGGTGGATTGTTATAAACGTGGTGGCGATATTTTACGTGGCCTGAGTTGCTATTATTCCGGCAATCCAGAAACAGGAACTAAACCTGAATCAGATTTTAATAACACCAGTTATATACAGCGCATAGGGTTTAATCCGCCTGATAACAAAAAAAACTGGGTGGTTCCTTCAGTTAAAGATGCAATCAGAAAGGAGAATGTAACACAGAGTATCAAACCTAAAGAAGTTACCGTATATCCCCAATATGCCATGCGTGGCACTGTTTTAGATGAAAAGGAAACAAACGATGTTAAAACTCAATAAACGTTATTTAACTCTTTCTGTATTTATGGCTGCATTGATGCTTTGTGTAGCAGAACCCGCCTTTGCTGATGATGTATCCACTAAAACAACTGGCTTCTTGCAGAAGATTATCGATTTTCTGACGGATATCCGTAAGCCAGCAATCACCATCATTGCGCTGGTAATTGGTTATATCGCTATCTTCTCACGCCAGCATGCAGCGTGGATTACGCCTTTAATTATCGGGATTATCATCTTTATTGTTGCGCCATATCTGCCTGACTGGTTAGCGTAATATGTAATAAAAGGGGGCGTAAAAATGAGTACCGTTTTTAAAGGGCTGACACGCCCCGCTTTGATTAGGGGGCTGGGCGTTCCGCTCTACCCCTTTCTTGGAATGTGCGTTATCTGCGTTTTGCTTGGTGTCTGGATTCATGAGGCTATGTATGCCCTCATCCTCCCTGGCTGGTATGCCATCAAGCGAGTTACGAAGATAGATGAACGCTTTTTTGACCTGCTTTATCTGCGAATGCAAATCAAAGGTAATCCTCTGGCAAACAAGCGCTTCAATGCCGTCCATTATGCGGGGAGTTCGTACGACGCAGTCGATATATCGAAAGTGGACAATTTTATGAAGCTTAAAGACCAGTCTTCTCTTGAAGAGTTAATCCCGTATTCATCACACATCACTGATAACCTCATTGTTACCAGAAACCATGATCTACTGGCGACCTGGCAGATTGACGGAGCTTATTTTGAATGTGTAGATGAAGCGGATCTAGCCTTGCTTACTGACCAGCTCAATACGCTGATACGTAGTTTTGATGGGAAACCCGTTACCTTTTATACGCATCGTATTCGGGTTAGAAAAGAAGTGCGACCTGTATTTGACAGCAAGATCCCATTCGTCAACAGAGTGATGAACGATTATTACGAATCGCTTTCGGCGGCTGAGTATTTTGAAAACAAATTATACTTAACCGTATGCTATAAACCGTTCAGCGCTGAAGATAAAGTGACGCATTTTCTTTCAAGGAAAAAGGGTAATAAAAATATCTTCGAAGAACCCATTAATGATATGAATGAAATATGTGGCAGATTGAGCACCTATCTCTCCCGGTTTCATTCCCGTCGTCTTGGGTTATATGAAGAAAATAATATAGTTTATTCAGAACAGCTTACGCTGTTCCAGAAACTGCTATCCGGGCGCTGGCAGAAAGTCAGAGTCACCAATAGTCCGTTTTATACATACCTGGGTGGTAAAGACCTGTTCTTTGGTAACGATGCTGGACAAATCACTGCCTCTGACCATGCCCGGTACTTTCGTTGCATCGAGATCAAGGACTACTTTCAGGAAACGGATGCCGGAATTTTTGATGCACTGATGTATCTCCCGGTTGAGTATGTACAGACTTCCTCTTTAACTCCGATAGACAAGCAGTCTGCAATCAAGGCGCTGGATGATCAGATCGACAAACTCGAAATGACCGATGATGCCGCTAAATCCTTGCTCGCTGATTTGAAAGTTGGTCTCGATATGGTCTCCAGCGGCTATATTTCTTTCGGGAAAAGTCACCAGACACTGATTGTTTACGCAGATTCGCCGGAGCGTCTGGTGAAGGACACCAATATTGTAACGACGACCCTGGAGGATTTAGGGCTGATCGTAACCTATTCAACCTTGAGTCTTGGCGCAGCGTATTTTGCTCAGTTACCTGGTAACTACACGTTACGGCCACGCCTGAGTTCTATCAGCAGTCTGAACTTTGCCGAAATGGAAAGCTTCCATAATTTCTTTACGGGCAAGGAGAAAGGAAATACCTGGGGTAACAACCTTATCACCCTGGGCGGCTCAGGAAATGATATTTATAACCTGAACTATCACATGACTACCGAGCATCAAAATTACTTCGGTAAAAACCCCACGCTGGGGCATACCGAAATTCTTGGTACGTCAAACGTGGGTAAAACGGTAGTGATGATGACTAAGGCTTTCGCCGCCCAGCAGTTCGGAACGCCGGAGTCTTTTCCACCTGAGAGGAAATTAAAGAAGTTAACTACAGTATTTTTCGACAAAGATCGGGCAGCGGAACCCGGTATACGTTCAATGGGAGGTGCTTACTTTCGGGTGAAAGAAGGAGAGCCGACTGGCTGGAACCCTGCGGCTTTGCCACCAACCAAACGAAATATTTCCTTTATGAAAGACCTGGTGAGGCTACTTTGTACGCTTAACAGTGAGCCGCTTGATGATTACCAGAACCGCCTGATTTCTGATGCCGTTGAGCGCCTGATGCAGAGGTCAAACCGCTCTTATCCGATCAGTAAGTTGCGACCACTCATTCTGGAGCCTGATGATACTGAAACCCGGCGGCATGGGCTTAAAGCACGCCTGAATGCCTGGGTGCAGGGAGGGGAATTTGGCTGGGTGTTTGATAACCCGGACGATACGTTTGATGTGGACAACCTGGACGTTTTCGGTATAGACGGAACGGAGTTTCTGGATAACAAAGTACTTTCCAGCGCCGCTTCATTCTATCTCATCTACCGTGTCACCATGCTGGCTGATGGCCGCCGACTCCTTATCTACATGGATGAGTTCTGGCAATGGATTAACAACGAAGCGTTCAGAGATTTTGTTTATAACAAACTCAAAACCGCGCGTAAGCTGGATATGGTGCTTGTCGTAGCGACACAATCGCCGGATGAACTAATTAAATCACCTATCGCGGCTGCCGTTCGTGAGCAGTGCGCAACACATATTTATCTGGCGAACCCCAAAGCGAAACGTAGTGAGTACGTAGACGAGTTGGAAGTGAGAGAACTTTATTTTGACAAAATTAAAGCCATTGACCCGTTATCTCGTCAGTTCCTGGTCGTCAAAAACCCACAGAGGAAGGGGGAAAGTGATGATTTTGCGGCTTTCGCAAGACTGGATTTGGGAAAAGCGGCGTATTACCTACCGGTTCTCAGCGCATCGAAACCCCAGCTTGAACTGTTCGATGAAATCTGGAAAGAGGGTATGAAGCCGGAAGAGTGGCTTGATACCTATCTGGAACGTGCAAACCTGATTTAAGGAATCCCTATGAAAAAGCATATCGTTGCGGCACTTATCGCCTCTGTTACGGTTATTTCTGGTGCTCAGGCTGGTGTTCCCGTTGCCATTGATGCAAACCCTGAATGGGCTGTCGAAGCGCAGCGCTGGACAGAACGCCTCAAACAGTGGCAGGACACCGTGAACCACTACCAGAAACAGATTAATGCCTATAAGCAGGAGCTGCTGACCAAAACGGGCATTCGTGATGTTCAGGGACTGGTGCAGTCTGCGCAATCTGTCAGTAAGGAGCTGGAGAATATTTACGACCAGGGGAACTCCTTCATTGATGATTACATTAAGAACCCGGAAGCCACCCTGTCTGAGCAAGCCAGATCACTACTGGCCGATTATAAGGTGACAAACACCTGTAAGGGGCTGGGATATACGGGCGACCTGGTGCGGGGCTGTGAAGCCTCGTTCCTGTCCCAACTGGCGGGGATCGAGTACGGCAATAAGCTGGAAAGTAAACTGCGTGAAGATAATCAGGAAATGGCCGACCTTATCGACCAGGTGAAGAACGCCCAGGACACGAAAGCCACGCAGGACGCGACAAACGCTGTATCTCTGGCAAGCCTGAAATTTAACAAGCTCAAATTTCAGTATGAAATGTATCGCGATAAGCAGCGCGATCTTGCAGAATACAAGGAGAAAATGTTACAGGCTGCTTTCCAGGAGAAACAATTAGGGGCTGTTAATAAAGAAACTCCAGTTGTTGATTATAAGGCAGCATTTGAGCAGCAGAGTTATGAAATGAACTGAGGGGGCATTTTGAAAAAATATGGATTAGTTGCGGCGTTGGGGCTTACATTCTTTCTTACCGGGTGTGAAGAGGTTAAATCTGTAGACTGGTGGCAGTCTCACGTAGACGATGCAACGAAAAAAGTAGCTGAATGCAAAAAGTCTGGGAGTGATAGCGAAAATTGTAAAAACGCTAAGGAAGGTTTGTTTAGGTATAAGCAATTACATCCTAAGCATGTTGACTATAAAGATGCATTCAAAAATGTATTGGGTAAAGATGGAAGTTAAAAGGAGCCACTATGGCAACTTCTAGGTATTTCCAGGCCGCTCATGACGTTATTGTTAAAACTCTTGACCAGTCTTTGACTGGTCAGTTAGATAACTTTTCTTCAATAGCGTCTCAGTTGGGTAAGTATGGTATTTCCATTTATATATTGTGGTATGCTTATACCACAATAGTTGGGAAGCAAAAAACCGCAGTGCAGGATTTTTTATGGAATCTATGTCGATTCTGGTTAATTCTTGTTTTTGTCAAAAATATGGGGGGGTGGTTAGATAGTGCAACTCAGGCCATTGATGGGCTGAAGGAAACATTTGCAGGAGGTGATCCTTGGAAATGGGTTGACGAGCTTTGGGAGAAAGTTCAGCAGGTAGCTGCATATCTTATGTCCAAAGATACTTCGAAGTATGTGAAAACAGATGGGGCAATCGCATCTTTATTAACATATGCTGGAGGGATAATTGTATTATTGCTAACTTCAATAGTTTATCTCGCCGCCGAAGTTACTATTAAGATACTTACTATTACCGCGCCGTTGTTTATTATTTGTCTTTCATTTGGTTTTTTAAGGCAGATGTTTAATAGCTGGCTTCAGTTAATATTTAGCTCATGCTTCATATTCTTGTTTTGTGGACTGGCAATTAAAGCGGGAATGACGTTCCTTAATGGCATTCTGACAATCTCGATAGCTAACGCAGATGAATTAAATCTGATATCAACAGGTGCTCAGGCCGGGGTTGCAGGTGCATTTATGGCCTGGATTATCTGGCAAGCCAAAACCTATGCTTCCCAGCTCGCAGGGGTAGGTGTTGAAGGCGCGATGCAAGGGGCAGCCGCAATGGGGATTGGTGCTGGCGTGTTCGGCGCTTCCCGTATGGGGCGTAATCTGCTTGGTATGGGTAAAAATGCAGGGATTGGCGCATGGAAAGGCGTTCGCCGTCAGGATGGAGGGTTCAACCAGTCTCCTGGTGTATCCGGTAAGTTGGGGAATCTGACCGGGCAGGGCGTTAATATTGGCGCTAAGAGGCTTCGCCAGGCTGCGATAGATGCGGCGAAAAAGAAATATGGAGGTTAACCGATTTATTATTTAAATCTGTCAACTCTGAGTATTCCGCAGTAAATATTCACTCCTCTATTTAGGTTCTCACATTATGAAACTACTTATCACGGCTATCGTGACGCTTTGCCTTGTCGGATGCCAGGCGTCACATAAACAACCTCCGGTATCCGGAAAAAGCGAACCTGTTAATTCAGCAGAGGTAATGCGCAATGGGGTTTAAACTCTCAGGATTAAAACTTCCCGGTTTTAACAAGAAAAAAGAAGTAACAGACTCTTCAACTACTTTTGAAGAGAAAAATGTATTGATGCAGGAGAAAATGAACCGCATTTATAAATTCGGTGGTATTGCAGGAATTGCCGTAGGTGTGTTGTCTCTGCTGGCACTGAATGGAGCGCTACCGTTAAAGAGTACGGTTGTCGAAGCTTATCTTATTAATGGTGTCACTGGCGTTGCTGAAAGACTGACTTCTGTTAAAAAAGAAAACCTCTCTGAAAATGAAGCGCTGGCGAAATACTTCATTACTCAGTATGTGAAGCGTCGTGAAGGGTATAACTATTTCAGTCTCCAGCATGATTATGATTATGTTCTGTTATACAGCGCTGAGAATGTGGCGACGGATTACAACGCTTTAATCAACGGCAACCAGTCACCAAAGGTTATTTATAATAAAGCGGAAAAAACCGCATCTGTTCAGGATAATCCGTCCGTGATTATCAGTCCGTCATCCCGGCGCGATGATAAGGATATGGGAGCATATATCCGGTTTAAATTAACCATTCGAAATGTCGCGACAGGCCAGGATGATTATGAATACTGGAACGTCCGGTTGACCTACCGTATTGAACCACAGGTCGAAATGGCATCGGGTGACCGAAACAATAACCCGCTTAAATTCGTTGTAACGAGCTATGTACGCGATAAAGAAGTGAAAGGGTGAAAACTATGAAAATGAATAAAGGAGCGCTAATTGTGGCGCTTCTGTTGGTGTCGCACGTCTGCCATGCAGCCGTTACGCCAGCAGCCAGCCGTTTTGATCCCCGTAATCAGATTGTCAGTTATAACCCGCAAAACACGACAGTCATAAACAGTGCGGTTGGCTATACAACCACACTGGTATTTGACGAGGATGAAACGGTCATCAGTGCCAGAACCGGCTTTCCCCAGGGCTGGGAAGTGAACAAAGAAGATAACCTGGTGTATCTCGAAGTGAAGCCCGTCAAACAGACCGTACAGAAGAACAGTACCGATGACAATGGCAACCAGTCTTCAGAGGTGGTCAGTGTAGCCCTTGATCCTGAAAACGACCTGGAACGCTGGCGAACAAACCTGTTTGTTCGCACAACAAAACGTAATTACAGCATGGAGCTGAACGCCCGAACGTTCCGGCAGCCGGATAAGATCGCGTTTGTTGTTAATTACCAGTACCCGCAGGAACGCCGGAAGGAGCAAGCCGAAATCGAGAAGAAACGGATTGAGGCTCTTAACAAGCGGCAGGAGGAGCAGGCAATCAACCGTTCTCTTGAAAATGCGAAGTCGCCCAAGAACTGGGATTACTGGAAGCGGGTAGCTGACGGCAGCCAGTCAATCAGCCCGGATTATGCGTATGACGATGGCCGCTATACCTGGTTTGGCTTCAGCCCCCTGAAGAAAATCCCCAGCGTCTTTGTTATGAGCGGCGAACAGGAAACGCTGACTAACCCGGTAGTCAAAAACAGCGGTAGTTTCACCGTTGTTGGTGTTCCTGTAGACCAGCGCTTTGTTTTGCGTATGGGAAATCAGGTTGTTGGGGTTGAAAACCGGGGCTTTGGCAAAGTCCGTCTTCCGGCAGGAGATACCGTTTCCCCGAACGTTGAGAAAGAGGTGATCCAGTGAGCGAACAGGAAAATAAAATCCCGACCGCTGTAGAAATTGAACAGGCACTACGGGAACGGCATCAGAAAGAAATTGAGCAAAACGATAAAACGGATAAGGGAGATGAAGATGACGGTAAATCAGTAAAACGCCTGGGTATCGAGAAGCTTAAAAAATCGCGTAAGGGGCTGGTGATTATTGTGGCCGCTTTCCTTCTGCTGGCCGCCGGGGTGTCTGTCTACTATATCCCGTCGATTATTCGTGCAATGTCCAGCGGTGATGATAAGCCTGCCAGTAATGCGGTATCTACTGGTTCAGTAAAACGCGCGACAGGACTCAGCGATGATGTTGATCCATTCAACACCAGGGAAGAACCGGCTAAGACGGAAGAACGGAAGGAAAGCAGCGGCAAAGCGGAAACGCCACCGGAAAAAGTACAACAAAACTTCAGTCGTGCGCTGGATGTGGCCTATGGGGGGAGCAGCTCTGCTTCCTCAAATTCCGGTGGTAGCTCATCAACGGCCAGCAATACCAGGAACGAGGGGGACGGCAGTGACAAACAGGCAGAGGTTCAGCCTGTTAATGCAGGGCAACCCGCGCAGCTTTCTAAAATTACGCGCGTTCCTTATGACCCTAACCTTTTCATTCCTGAAAATACCGCGATTAAGTGCTCGCTGGACAGACGATTTATTTCTGATCTGGCTGGCAAGCTGGTCTGTACGATTAATGAGGACGTATACAGTGCAAACAGGAATGTAAAGCTAATTGAAAAGGGAACAGCCGCATATCTGATGTATAAAACGGGCACGTTCAATCATGGTCAGGGAGCCGTGTTTATCGCTGCTACGAAGCTTAGAACACGTAAGGAACCTTTCATTGATATTCCGCTGATTGATACACAGGCGGCGGGAGCGCTGGGTGAGGCCGGGGCTTCAGGCTGGATTGACACACATTTTGCAGATCGCTTTATGGGCGCGATGATGGTTGGCATGATCCCCGATGTGGCACAGGCCGCAAGTGGAGCGGCTAAAAGTAATAAAGACAATCAGACCGACTATACAGCTAACAGTCGCCAGGCTTTTGCTGATATAGCACGCGAAGCGTTTGCAAACAGCGTAAATATCCCTCCAACGCTTTATAAAAACCAGGGGGAAATCATTACTCTGATCGTTGGTCAGGATTTGGATTTCTCCAGCATCTACAAATTAAAATGGTGGGTACCCGGAGGTAATTTGATAACGCTGGATATCAAAGAGTTTTCAATGCTGTTAGGAATAAGGGAAAGCGAAATATACCATCATATTCGTAAGGGCATACCAATAAATGGCGTTCCTTTCCCCAAACCACTGAAACAAATCAAAACGCATCGATTTAATTACGAAGAAGTCATGCGTTTTATTGAAGATCTTAAAGGAAAAGGTGAATTATGAAGGATGCAGAGAACAGACATATTGTTTATGAAATAATAGATGAATACTTCCACCACTGGCTTAATGAAGTCGATGGTTTAACTGAAATCGCGGTAAATCGGCCTGATGAACTGTTTGTTAAAGTGAGTGGGAAATGGCAGCAGCATGAATTAAAAATGGATTTTAAAGATTGTATGTCATTTGCAGGGGCAATTTCTGATTATCATGATGGTGGGTCGGTTACGCCAGAATATCCCTTACGTTCTGTTACGCTCCCCGGAGGGGAGCGTGTACAAATAGTTATTCCCCCGGCAACGGAACGGGAAACGGTGTCTATTACAATCCGTAAGCCGTCCAGTGTGTTTATCGACCATGATACCTTTGTTAAACAAGGGTTTTACTCAAGGCTAAATCAAGGGGGGAAATTCAGGGATAAAGAAGATGAATTATCTTCAATGTTTAAAGATAATTGTTTTGAACAATTTGTCCCTGAATGCCTGGTTAAGGGTAAAACGATGGTATTTTGTGCCGGAACGGGGGCGGGTAAAACCACCTTTGCAAATGCCTGTCTTCAGTACATACCCCATCATTTACGATGTATTTCAATTGAAGATACAGATGAAGCAAAGTTCAGGTTCCACAAAAATCATGTGAAATTGTATTACCCGTCAGAAGGGGAAAGTAGCACTGTCAGTTCCGCTTCTCTTTTACGTTCTGGTTTTCGTATGAACCCGGACAGAATCCTCATGACTGAGGTTCGCGGCGCGGAAGCCTGGGATTTTCTCAAAGGCTCAAGTTCCGGCCATGCAGGGAACCTTACAACCGTCCATGAGAGCACCCCTGAAGATGCTGTGCTTGGACTGGTTCAACGGTGCTATATGAACCCTGAATGTCAGAATTTGCCTTATAACATTATATTGCGGAGGGTGTTAAGCAACGTCGATGTGATTATGAGTATTAAGTACATCGATGAGGAAGATAACCGATTTGCATCGGGTATTTACTATCGTGATATTCATTTTCAGGAATACTTTGAAAAGCTGAAGGAGTGATTATGTCTCTGAAACTACCCGATAAAGGCCAGTGGGCTTTTATCGGTCTGGTTATGTGTCTTGTGACGTATTATACTGGCTCAGTTGCTGTTTACTTCCTGAACGGAAAAACGCCGCTTTATATATGGAAGAATTTTGATTCCATGCTGCTGTGGCGGATAATAACAGAGAGTAATATACGGACAGATATCAGGTTAACCGCAATCCCCTCTCTTTTATCAGGTATGGTTTCGTCTCTCATTGTGCCTGTTTTTATTATCTGGCAACTGAATAAAACGGATGTTGCTCTTTATGGTGACGCGAAGTTTGCCAGTGATAATGATTTAAGAAAATCGAAACTTCTGAAATGGGAGAAAGAAAACGATACTGATATTCTTGTAGGTGCATATAAAGGTAAATACCTGTGGTATACCGCACCAGATTTTGTATCACTTGGCGCAGGAACCCGCGCAGGTAAAGGGGCTGCAATAGGTATTCCAAACCTGCTGGTCAGGAAACATTCCCTGATTGCATTAGATCCAAAACAGGAACTGTGGAAAATCACCAGTAAGGTACGTGAAAAACTGCTGGGTAATAAGGTCTATCTTCTTGACCCTTTCAACAGTAAAACGCATCAGTTTAACCCGCTTTTCTATATTGATTTAAAAGAAGAGAGCGGGGCTAAAGACCTGCTGAAGCTGATTGAGATCCTGTTCCCGTCTTACGGTCTGACCGGAGCGGAAGCACACTTTAATAACCTCGCGGGGCAGTACTGGACGGGGCTGGCAAAATTGCTTCATTTCTTTATTAACTACGACCCCTCCTGGCTTGGTGAGTTCGGGCTTAAACCTGTTTTCTCAATTGGTTCTGTAGTCGATTTGTACAGCAACATCGACCGGGAGCTGATACTCAGTAAGCGAGAAGACCTGGAGGGAACAAAAGGACTTGATGAAAACGCGCTGTATCATCTGCGCGATGCCCTGACCAAAATCAGGGAGTATCACGAAACCGAAGACGAACAACGTTCGAGTATTGATGGTTCGTTTCGTAAGAAAATGAGTCTGTTTTATCTTCCGACTGTTCGCAAATGTACAGACGGCAATGATTTCGACCTTCGCCAGTTGCGCCGGGAAGATATCACTGTTTATGTTGGCGTTAATGCTGAAGATATGTCCCTGGCCTATGACTTCCTCAACCTGTTCTTTAACTTCGTTGTGGAAGTCACGTTACGAGAAAACCCTGACTTTGACCCGACACTGAAACATGACTGTCTGATGTTCCTGGATGAGTTTCCGTCTATTGGTTACATGCCAATCATTAAAAAAGGTTCGGGTTATATTGCCGGGTTTAAACTCAAACTTCTGACGATTTACCAGAATATCAGTCAGCTTAATGAAATCTATGGTGTTGAAGGTGCCAAAACGCTGATGAGTGCGCATCCCTGCCGGATTATCTACGCTGTCAGTGAAGAAGATGACGCTAAAAAGATTTCGGAAAAGCTGGGCTATATCACCACCAAATCGACAGGTTCAAGCAAGACAAGCGGACGCTCAACGTCGAAAAGCAGTTCAGAAAGTGAGGCGCAGCGCGCCCTTGTGCTCCCGCAGGAGCTGGGAACGCTGGATTTTAAAGAAGAATTTATCATTCTTAAAGGTGAAAACCCGGTTAAAGCTGAGAAAGCCCTCTACTTCCTCGACCCGTATTTTATGGACAGGCTGATGATGGTAAGCCCCAAACTTACCGAGTTAACCGCCTCTCTGAATAAGACTAAAAAAGTATTGGGTGTTAAAGGGCTTAAATATCCTTCTAAAGAAAAAATGCTCTCCGTCGGTGAGCTGGAGTCAGAGGTTTTACTATGAAAAAATGTTAATGGTCAGTGTCCTTTTTCTCTCTGCCTGTTCCTCTCCGCCTGAACCACCACAGGTTGACTGGGAACAGAATCCTGAAACGGTGAATACACAATTAATGGACTGGCAACCGACATACTCAGTTATTAAATCAGACAAGGTTAATTCATCGTGGGTTAAGGTTATCCATAATTTCAGGCCTGAAAACCGCCTTTACGATGATGCTGTTTTTTATTCTGTTGCACATTCTGATTCAGTGATTGTTGAAACCAGTAACGGAACAGACTTTTTCACAGCTAAAAACTGGTTGAGGGCGAACGGTGCAAACGGTGTCATACAGTATCGTTATAAAATGAACTGTTTTTCTTGCCGGACTACCAGTGTTTATTTATCACGATAACGCCGGATTAATAAAATTCTTGTAATTTTATTGAGCCGGGGTTGAACAACCGCAGGGCGTTAGTTGCCCGTTATCCATGAGGTGTATATGAAAAAGACCGTAACCACGGCAGTGCTGCTTTGTGCGTTCGCTGCCGGAACTGCTCATGCTGAGGAAAAGGCCGACCCGAACGATCCATGTGCAATGGTGTTATGCCTTGCCGGAAAACTTGACGGAAGTAGTCCGGCAGAATGTGACCCGATGTATAAAAGTTTTATGTCAATCAGGAAAAAGAATAAACATGGTTTTTTACCCGATCACACGGCAGATGCCAGAAAGAAAAAACTAAATGAATGTCCTGCGGCTGATGCAGGTACAGTAAGTAAAATTATTTCAAGCTTTGGCCGATTGAAAAACTTTTAATTCTGTTTGATTTAATTCTGATGTGAATACGCAAGTTTCAGGTTTGAGACTGGAACTTGCGAACATTTTTTACAGGAAGGGAGGATGAAAATTATTAACGTACCTGATGCAAGAAGTGTGAGAATTGCCGGAATTGAAATTATCGATAGTGTCAAATCGGTCGCTTCTGTTTTTACTGAAACGGGGTTGTTTATGGTCTGGTTTCCGGCAATTGTCTATGGGCTTTATCGTTTTGTTATCGTTTTGTGGGATGCTTCCTTAAAGCCGCAATCGCTCAATGCAGTTCATTTACGGCATGGCACAGATCTCTGATCGTACCTATGATCAGCTCAGCACTATCCAGGCGAACTATCTTCGCCTGGAAACATTGAATGCTGCTAACGACAACATCAATAACGCGACAGCCAAGCTACCCATCTTCCGGTACTACAACATCCAGGAAGATGTGATCCACGCCAGTGCCGATGGTCAAAAGTTCGAAGCCCGGCGCGAGACCTTCAAAACCCGTTATTCGTCGAAGTACTTTGGCACTCAAAAAGGTGTTTCTGCCATGACCTTGATCGCCAATCACGCTGCGATCAACGCCAGAGTGATCGGCGCCAACGAACATGAATCGCACTACATCTTTGATTTGTTGATGAGCAATACGTCAGACATCATTCCGGATGTGCTCTCAACCGATACCCATGGGGTGAACCATGTGAACTTCGCGTTACTGGATCTGTTCGGATACCAGTTTGCCCCACGCTATGCCCAGGTTGGCAAAGTGATCAATGACATGTTTGATGTCAAGGAAGACAAAGAACACCGAATTCAGCTGTGCTTAAAAAAGCCAATCAATACCCATCGTATTGCGCAGCACTGGGATACCATCCAACGGATTGCAGTATCACTTAAGCAGCGGAAAACAACGCAAGCCACCTTGGTGAGAAAGCTCTCGGAGTACAAGCGCAATCACCCGCTGCTGGAAGCCCTGACGGAATACAATCGCCTGGTGAAAGCGAATTATCTACTGTGCTACATCGATGATGCCAGTTTAAGAAACTATGTTCAGCGCGCGCTGAACCGGGGAGAGGCCTATCACCAACTGCGTCGGGCCGTGAGCAGCGTCAATGGGGATCAGTTCCGGGGCAGTTCAGACGAAGAAATCCAGCTATGGAATGAGTGCGCTCGCCTGGTCACCAATGCCATCGTTTACTTCAACTCCAGGATACTCAGCCAGCTGTTGACCAGCTTCGAATACCAAGGAGATACCAAGAGAATAGATATCGTCAAACAGGCATCCCCTGTGGCCTGGCACAACATTAACCTCAAGGGGACTTACCACTTCGAATTGAGCGAAAAATTGCCAGATCTGGAGGAGCTTATGCGCTCAATCGAGGGATATTTACCCGTCAGCGAAAAGTAATACCCCTCTGGAGGCCACGGACGGCGCGGCCTCCAGAGCACTTTGTCGTTTTTGGACGGAAAATCCCTAGAACCCCATTATCTAAAAATACTTAATTGTCTTTTAACGTCGCTAAATTTTAAATAAATAAGTGAAGAGTGTTAGTGGAGCCACTGATTTAAAGTTGGCAGAGTAAAACTTGAAGTGCGACATAAACCACCTAATTAATTTAAAGGGTTTATGGAGTATATAAAATTGTCATACCATCATCTTAACTTTGAAGATCGTACTGCATTAATGCTTGAGTCAAGAAAAGAAGGCTTTTCAGCCAGAAAATTTGCTGAACTCATTAAAAGACATCCTAGTACGATCTATCGTGAGCTTAAAAGAAATAGCATCAATGACGTTTATCAAGCTCGATATGCTTCTGATAACACCTTCGCTAGACGTAGACGTGGTCACAGAAAACTCAAAATCGATTCAATCCTCTGGAAATTTATTGTTGAAGCGATCCGTTGTTTATGGTCTCCTCAGCAAATAGCAAAGCGTTTAAAGACATTTCCTGATTTGGATCAAACAATGAATGTAAGCCATACAACGATTTATTCAACGATACGAGCATTACCAAAGGGTGAGTTGAAAAAAGACTTATTATCCTGTCTACGTCATGAAAATAAAAAGCGAAAAGCTAACGGTGAACCTAAAAAAGATTCTATATTACAGGATATTAAAACTATTCATGAGCGCCCAGCCGAAGTTCAAGAAAGAAAAATACCGGGTCATTGGGAAGCTGATTTAATTAAAGGTAAAGACAATAAAAGTTCGATAGCAACACTTATTGAACGAAATACACGGCTCTGTATCTTGGCAACATTACCTGATGCAAAGGCAGAATCAGTGCGCAAGGCTTTAACTGAAGCTCTGAAATATTTACCTGCAGAACTGCGTAAAACGTTGACCTATGACCGTGGACGTGAGATGTCAGAACATAAAATACTCGAAGAAGATTTAGGCATAGATGTATATTTCTGTGACCCACATTCACCCTGGCAAAAAGGCACATGCGAAAATATGAATGGTTTAATTAGGCAATATTTACCTAAGGAAGGTGCGAACAAGTCCCTGATATGAGATCATGTTTGTCATCTGGAGCCATGGAACAGGGTTCATCATGAGTCATCAACTTACCTTCGCCGACAGTGAATTCAGCAGTAAGCGCCGTCAGACCAGAAAAGAGATTTTCTTGTCCCGCATGGAGCAGATTCTGCCATGGCAAAACATGGTGGAAGTCATCGAGCCGTTTTACCCCAAGGCTGGTAATGGCCGGCGACCTTATCCGCTGGAAACCATGCTACGCATTCACTGCATGCAGCATTGGTACAACCTGAGCGATGGCGCGATGGAAGATGCTCTGTACGAAATCGCCTCCATGCGTCTGTTTGCCCGGTTATCCCTGGATAGCGCCTTGCCTGACCGCACCACCATCATGAATTTCCGCCACCTGCTGGAGCAGCATCAACTGGCCCGCCAATTGTTCAAGACCATCAATCGCTGGCTGGCCGAAGCAGGCGTCATGATGACTCAAGGCACCTTGGTCGATGCCACCATCATTGAGGCACCCAGCTCGACCAAGAACAAAGAGCAGCAACGCGATCCGGAGATGCATCAGACCAAGAAAGGCAATCAATGGCACTTTGGCATGAAGGCCCACATTGGTGTCGATGCCAAGAGTGGCCTGACCCACAGCCTAGTCACCACCGCGGCCAACGAGCATGACCTCAATCAGCTGGGTAATCTGCTGCATGGAGAGGAGCAATTTGTCTCAGCCGATGCCGGCTACCAAGGGGCGCCACAGCGCGAGGAGCTGGCCGAGGTGGATGTGGACTGGCTGATCGCCGAGCGCCCCGGCAAGGTAAGAACCTTGAAACAGCATCCACGCAAGAACAAAACGGCCATCAACATCGAATACATGAAAGCCAGCATCCGGGCCAAGGTGGAGCACCCATTTCGCATCATCAAGCGACAGTTCGGCTTCGTGAAAGCCAGATACAAGGGGTTGCTGAAAAACGATAACCAACTGGCGATGTTATTCACGCTGGCCAACCTGTTTCGGGCGGACCAAATGATACGTCAGTGGGAGAGATCTCACTAAAAACTGGGGATAACGCCTTAAATGGCGAAGAAACGGTCTAAATAGGCTGATTCAAGGCATTTACGAGAGAAAAAATCGGCTCAAACATGAAGAAATGAAATGACTGAGTCAGCCGAGAAGAATTTCCCCGCTTATTCGCACCTTCCCTAAAGGGATTGATTTAAATCAGGCAGATCAGCATTATTTAAATCAAGTTGCCATGTCACTGAATACTCGTCCTAGAAAGGCGTTAGATTGGCTTACACCATTAGAGAAATTTGCTCAGCTTGTTGATTATCATATGGCTTTTGAAACTGTCGCACCTCATGTTTGAATTCGCCCCATATTTTTGCTACAGTGAACCAAATTAAGATCATCTATTTACTAGGCCTCGCATTTGCGGGGTTTTTAATGCTGAATAAAAGGAAAACTTGATGGAATTGCCCAATATTATGCACCCGGTCGCGAAGCTGAGCACCGCATTAGCCGCTGCATTGATGCTGAGCGGGTGCATGCCCGGTGAAATCCGCCCGACGATTGGCCAGCAAATGGAAACTGGCGACCAACGGTTTGGCGATCTGGTTTTCCGCCAGCTCGCACCGAATGTCTGGCAGCACACTTCCTATCTCGACATGCCGGGTTTCGGGGCAGTCGCTTCCAACGGTTTGATCGTCAGGGATGGCGGCCGCGTGCTGGTGGTCGATACCGCCTGGACCGATGACCAGACCGCCCAGATCCTCAACTGGATCAAGCAGGAGATCAACCTGCCGGTCGCGCTGGCGGTGGTGACTCACGCGCATCAGGACAAGATGGGCGGTATGGACGCGCTGCATGCGGCGGGGATTGCGACTTATGCCAATGCGTTGTCGAACCAGCTTGCCCCGCAAGAGGGGATGGTTGCGGCGCAACACAGCCTGACTTTCGCCGCCAATGGCTGGGTCGAACCAGCAACCGCGCCCAACTTTGGCCCGCTCAAGGTATTTTACCCCGGCCCCGGCCACACCAGTGACAATATCACCGTTGGGATCGACGGCACCGACATCGCTTTTGGTGGCTGCCTGATCAAGGACAGCAAGGCCAAGTCGCTCGGCAATCTCGGTGATGCCGACACTGAGCACTACGCCGCGTCAGCGCGCGCGTTTGGTGCGGCGTTCCCCAAGGCCAGCATGATCGTGATGAGCCATTCCGCCCCCGATAGCCGCGCCGCAATCACTCATACGGCCCGCATGGCCGACAAGCTGCGCTGAGCCATGGCTGACCACGTCACCCCCAATCTGCCATCGCGCGATTTCGATGTGACAGAGGCGTTTTATGCGAAGCTGGGCTTTGCGACGAGTTGGAAGGATCGCGGCTGGATGATCCTGCAGCGCGGCGGTTTGCAGCTCGAATTCTTCCCCTATCCTGACCTCGACCCAGCTACGAGCTCGTTCGGCTGTTGCCTGCGGTTGGATGATCTCGATGCCATGGTGGCATTGGTGAACGCGGCGGGAGCCGAGGAAAAAAGCACCGGCTGGCCGCGCTTCAAAGCTCCGCAACTGGAGGCGAGCGGCCTGAGGATCGGCTACCTGATCGATCCCGACTGCACGCTGGTGCGGCTGATCCAGAACCCCGACTGACCGCATGCCCGCGAAAATCAAGATTTGCGGGATCAGCACACCCGAGGCGCTCGATGCGACCATCGCGGCGCGGGCGGACTATGCCGGGTTGGTGTTCTATCCAGCGTCGCCCCGTGCGGTTACGTCGAATGTCGCGGGCGCTTTGACATCGCGCGCAGCTGGCCAGATCGCCATGGTCGGTTTGTTCGTCGATGCGGATGATGCTGTCATCGCCGACGCACTGGTGGCAGCCAAGCTGAACGCGCTGCAGCTGCACGGTTCGGAATCGCCCGAACGCGTGGCCCAGTTGCGCGCGCGGTTTGGCAAGCCGGTGTGGAAGGCGCTGCCCGTCGCCAGCGCCAGCGATGTCGCACGCGCCGCAGCCTATGCCGGGGCGGCGGACTTGATCTTGTTCGACGCCAAGACCCCCAAAGGCGCGCTGCCCGGCGGCATGGGGTTGGCGTTCGACTGGTCGCTGCTGGCCGGATATCGCGGTGCCTTGCCGTGGGGGCTGGCAGGCGGGCTAAATCCGACGAATGTTGCCGAGGCGATTGCGCGCACCGGAGCGCCGCTGGTCGATACCTCCAGCGGCGTCGAAAGCGCGCCGGGCGTCAAGGATACCGACAAGATTACCAATTTCGCCTTTGCGGTGCGCTTGGCCTAAATCGCGTCGATCAATAGGCGTCGTTCAGCGCAAAGATCGGCTTGCGGGTGCGCCACTGCCCTCGGGTGAAGTCGGGAAAATCTAACGTGCGATTGCCCTCAGCAATCGATTGTTCCGACAGAGGCGTGATCGCGCTCCAGGCCAGCGCGTCGTAAATGTCGATTGGCATCGGGGCCTTGGCCTTCAGCGCCTCGACAAAAGCGTGGATCACGAACCAGTCCATCCCGCCATGCCCGGCCCCTGCCGCCAGATCGGCGTAGCGTTTCCATAGCGGGTGATCGTATTTCGCAAACCAGCCCTCGGCAGGCTCCCAGCGGTGCGGCTGTGGGCTCTTGCCCTCCAGATAGATCGACTTGTTGACGTCCATCCACAGCCCCTCGGTGCCTTGCACCCGAAAGCCGAGAGAATAGGGGCGCGGCAGCGAGGTGTCGTGGCACAGCATGATCGTTTCACCATTAGTGCAGCCGATCATGGTGTTGACCACATCACCCAGTGCGAATTTCACCTCGGCGTTGGGATGATCGGCAGAGCCGTTCTTGACGACATAATCATGCAGCCCGCGCGCCTTACAGCCGAAGCCGCCAGCGCCCGCTTCGCCCGGCAACGCGACCTTCAGGGTGCGGGTCTGCGGCGGGTAGCACACGCCGGCATCGGCGCAGCCCTGGTACTTCACGGTCAGGGTGGTCGCGCTCGCGCCGGCCGCGGGCGTGCCGGTGAGGGTGCCGAGCAATTCCTTGCGGTAGGTTTCGACGTCGCCGAAGAATTCGTCGCGGTAGGCCTTGCCCTTCGGCAGCGCCATGGTCGCGCCGGTGAAGGCGGCATCGGCCTTGACCGAGGTGCGGTGCCGGTACAGGTAATAGCCGTCGGCGATCCGCCAGCGCACCTCGATGCGGTCCGGCGCGGTGGCCTGCGCGGACAGGACGAAGACCTCGTCGACCGGCGGCAGTTCGAAGTCCTGGGCGACGGCCGAGGTCGCGGGCAGCGCAAGCAGCAGGGCGAGCCCGGCCAGCCAGCGGCGCAGGCGGATCGTGGATGCGGTCATTGGCTCAGTTTACCGGTCGGCTCTCGGCGGCCAGCCATTGCAGGTATTCGGGCAGGCCGGACGCGGCTTCGACCGCGAGCAGCTCCGGGAGTTCGTAGGGATGCAGTTGGCGCAGGCGTTCCTGCAGGGCGGGGTAGGCCTCGGCACTGGTCTTGACCAGCAGCAGGACCTCGGCCGCGGCCTCGACCTTGCGTTGCCAGCGATAGACCGAACGCAGGCCGGGCAGGAGGTTGACGCAGGCGGCCAGGCGCTCGGCCACCAGCGCGGTGGCGATGCGCTCGGCGCTGTCGGCGTCGGGACAGGTGCAGAAGCAGATCAGGGCGCTCACCGGCATAGGGTAGCGGCTGCCCCGATCCGGCGGGCCTGGCGGACATCCGCGTGCGGCCCTTGAAAGTCGGCGGGCCCGCCCCATCTCGGTGGCATGCCGGGTTCGCCCGGTTCTGTTGTCCGCGGTTTGGCACTCGCTTCGCGCGACTGCTAAAATCGCCGGGTTTTTCCACGTCAATCAACCATTTACCGAGGTTGCCATGTCCAATATCAAGCCGCTGCACGACCGCGTGGTCATCAAGCGCATGGAAGAAGAGAAGCTGTCCGCCGGCGGGATCGTGATCCCGGATTCGGCCACCGAGAAGCCGATCAAGGGCGAAGTCGTCGCCGTCGGCACCGGCAAGGTGCTGGACAACGGCCAGGTCCGCGCGCCGCAGGTCAAGGTCGGCGACAAGGTGCTGTTCGGCAAGTACAGCGGCACCGAAGTGAAGCTGGACGGCGTCGAGCTGCTGGTGGTGAAGGAAGACGACCTGTTCGCGATCCTCGGCTGATCGCGCGTCGCTCCCACACATTTCTCATCCGAATAATTTTTCGAGGTAATTCGCAATGGCTGCCAAGGACATTCGTTTCGGCGAAGACGCGCGCTCCAAGATGGTGCGCGGCGTCAACGTGCTCGCCAACGCCGTGAAGGCGACCCTCGGCCCGAAGGGCCGCAACGTCGTGCTGCAGAAGAGCTACGGCGCGCCGACCATCACCAAGGACGGCGTCTCCGTCGCCAAGGAAATCGAACTGGCTGACGCGTTCGAGAACATGGGCGCGCAGATGGTGAAGGAAGTCGCTTCCAAGACCTCCGACAACGCCGGCGACGGCACCACCACCGCCACCGTGCTGGCGCAGGCGTTCATCCGCGAGGGCATGAAGGCGGTCGCCGCCGGCATGAACCCGATGGACCTGAAGCGCGGCATCGACCAGGCGGTGAAGGCCGCGGTCGGCGAACTGAAGTCGCTGTCCAAGCCGTCGTCGACCAGCAAGGAAATCGCCCAGGTCGGCGCGATCTCCGCGAACTCGGATGCCAACATCGGCGACCTGATCGCGCAGGCGATGGACAAGGTCGGCAAGGAAGGCGTGATCACGGTCGAGGAAGGCAGCGGCCTGGACAACGAACTCGACGTGGTCGAGGGCATGCAGTTCGACCGCGGCTACCTGAGCCCGTACTTCGTCAACAACCAGCAGTCGATGTCGGCCGACCTGGATGATCCCTTCATCCTGCTGTACGACAAGAAGATCTCCAACGTGCGCGACCTGCTGCCCGTCCTCGAGGGCGTGGCCAAGGCCGGCAAGCCGCTGCTGATCGTGGCGGAGGAAGTCGAAGGCGAAGCGCTGGCGACCCTGGTGGTCAACACCATCCGCGGCATCGTCAAGGTCTGCGCGGTGAAGGCCCCGGGCTTCGGCGACCGTCGCAAGGCGATGCTGGAAGACATGGCGATCCTGACCGGCGGCGTGGTGATTTCCGAGGAAGTCGGCCTGTCGCTGGAGAAGGCCACCATCAAGGACCTCGGCCGCGCCAAGAAGATCCAGGTGTCGAAGGAAAACACCACCATCATCGATGGCGCCGGCGAAGGCGCGGGCATCGAGGCGCGCATCAAGCAGATCAAGGCGCAGATCGAGGAGACCTCCTCCGACTACGACCGCGAGAAGCTGCAGGAGCGCGTGGCCAAGCTGGCCGGCGGCGTTGCGGTGATCAAGGTCGGTGCCGCCACCGAAGTCGAGATGAAGGAAAAGAAGGCGCGCGTCGAAGACGCCCTGCACGCGACCCGTGCGGCCGTCGAGGAAGGCATCGTCCCGGGCGGCGGCGTCGCCCTGATCCGTGCCAAGGCGGCGATCGCCGGCATCAAGGGCGTGAACGAAGACCAGAACCACGGCATCCAGATCGCCCTGCGCGCGATGGAAGCCCCGCTGCGCGAGATCGTGACCAATGCCGGCGATGAGCCGTCGGTCATCCTCAACCGCGTGGTCGAAGGTTCGGGTGCGTTCGGCTACAACGCCGCCAACGGCGAGTTCGGCGACATGATCGAGTTCGGCATCCTGGACCCGACCAAGGTCACCCGCACCGCGCTGCAGAACGCCGCGTCGATCGCGGGCCTGATGATCACCACCGAAGCGATGGTGGCCGAGGCCCCGAAGAAGGACGAGCCGGCGATGCCGGCCGGCGGCGGCATGGGCGGCATGGGCGGCATGGATTTCTAAGCCCCGCGATCCATCAAGCAAGACCACAAAGCCCGGCCTCGTGCCGGGCTTTGTGCGTTCTGGCGTCCGAGGCGGGAGACTTCCTACCCGCCCCGCGGCAATGTCTGACGCGAAGATCAGAAAACGCCGATATGAACGCGTGCTCGCGGGCGCAACCCTGAGCAGCCGTCCCTGCAACGGAGCGCTGCGTGCCGCGCCTGACCGCACCCCGGCGGCAGGCCGAGGTGTGCGCGCCACTGCCGGCCGCCCACGCCGCTGCGCGTTACGCGCGCCACCTGCCCGAGCGCACGCTGCTGTACGCGCTAGTGCAGGCGCACTACCCGGACTTCATCGCGCGTCTTGAGGCCGAAGACCGCCCGCTGCCCGAGTATGTGCGCGAGGAGTTCGAGACCTACCTGCGCTGCGGCGTGCTCGAGCACGGCTTCCTGCGCGTGGTCTGCGAGCACTGTCGTGCCGAGAGGCTGGTGGCGTATTCCTGCAAGAAGCGCGGGCTGTGCCCGAGCTGCGGCGCACGGCGCATGGCCGAGTCGGCGCGGCATCTGGTGGACGAGGTGTTCGGCCCGCGGCCGGTGCGGCAATGGGTGCTGAGTTTCCCGTACCCGTTGCGCTTCCTGTTCGCCAGCAAGCCTGAGGCGATCGGCCCGGTGCTGGGCATCGTGCATCGTGTGATCGCCGGTTGGCTTGCCGATCAGGCCGGCGTGCCGCGGGATACGGCGCAATGCGGCGTGGTGACCCTGATCCAGCGCTTCGGCAGCGCGCTGAATCTCAACATCCACTTCCACATGCTGTGGCTCGACGGCGTGTACGAGGACACCACCGAGCGTCCGCAGCGCAAGCCGCGCCTGCACCGCACCCGTGCGCCCACATCGGCGCAACTGACGGAACTGGCCAACACCATCGCGCATCGCGTGTGCCGGCACCTGTCGCGCCGCGGCTGGCTCGAAGGCGAAGACGAATCCGTGTTCCTGTCCGACAGCGCGGGTAGCGACGACGGCATGGATGGGCTGCGGATGAGTTCGATGACCTACCGCATCGCCACCGGTCGCGACGCTGGCCGCAAGGTCGTCACGCTGCAAACGCTGCCTGGCGACGCCGGTCCGCTGGAGGGCGACGCCGGCAAGGTCGGCGGCTTCTCGCTGCATGCCGGCGTGGCCGCGGAAGCACACGAAAGCCACAAGCTCGAAAAGCTGTGCCGCTACATCACGCGCCCGGCGATCAGCGAGCAGCGGCTATCGATCTCGCCACAGGGCAGGGTGCGTTACCAGCTCAAGACGCCGTGGCGCAATGGCACCACGCATGTCGAATGGGATGCGGTGGACTTCATCGCCAAGCTGGCGGCACTGGTCCCGCCGCCACGCGCGCATCTCACCCGCTTCCACGGCGTATTCGCCCCGAATGCAAACCTGCGCGCGCAGCTGACGCCCTCGGGGCGCGGCAGGCGGCCTGCGGGCGATGCGGCGCCAGTGGACGTCAGCGCCCACGACGAGCCGCGCAGCCCCGAGCAGAAGCGCCGTGCGATGAGCTGGGCGCAACGGCTCAAGCGGGTCTTTTCCATCGACATCACCACCTGCGCCCACTGCGGCGGCGCGGTGCGGATCGTCGCCAGCATCGAAGACCCCAAGGCCATTCGCGCCATCCTCGCCCACTTCGAGAAACACGGCGCGCTGGAGCAAGCGCACTACCGGCCCGCAGCGCGCGCCCCGCCGCCCGCCGCGTGATGAGGCGCCGGCCACACAGCCGGCAGCCAAGCCAGAGTCCGATCCGATGCGGCCACGACCCCGCAGGGCTGCGCTCGGCCCTGTGCCGGGATTCGGTGAGAAATGGCTACGCACTGAGCCGCTGCGTGGCCCCGCGATGTCGAAAACCCACGCATGAACCCCCGATCTGTGCCCGATCTGTGCCCAAAGCGGCGCTTGCGCGGCCGCTTCCTACCCGCCAGACTCGCCAAAAAGGGCGGTTGAACTTCCTATACCCATATCCCACTTTAGGTTTGGCAGCACCTTTTCAATCAGGTGGTTGTTTACACCAATCCAGCTATTGGCCAATTGAATATTGGTTTCGTCTACTTTTTTTGATTTTTTAAACAGCTTGGTAAAACCGAGTGCCAAAACAGGGGGACCATGTAAAAACGTGCTTCCTGTAATGACTGAACCAACTGTGAGTCCTTTACTGACTTTTTGCGGCACTGTTGCAAAGTTAGCGATGAGGCAGCCTTTTGTCTTATTCAAAGGCCTTACATTTCAAAAACTCTGCTTACCAGGCGCATTTCGCCCAGGGGATCACCATAATAAAATGCTGAGGCCTGGCCTTTGCGTAGTGCACGCATCACCTCAATACCTTTGATGGTGGCGTAAGCCGTCTTCATGGATTTAAATCCCAGCGTGGCGCCGATTATCCGTTTCAGTTTGCCATGATCGCATTCAATCACGTTGTTCCGGTACTTAATCTGTCGGTGTTCAACGTCAGACGGGCACCGGCCTTCGCGTTTGAGCAGAGCAAGCGCGCGACCATAGGCGGGCGCTTTATCCGTGTTGATGAATCGCGGGATCTGCCACTTCTTCACGTTGTTGAGGATTTTACCCAGAAACCGGTATGCAGCTTTGCTGTTACGACGGGAGGAGAGATAAAAATCGACAGTGCGGCCCCGGCTGTCGACGGCCCGGTACAGATACGCCCAGCGGCCATTGACCTTCACGTAGGTTTCATCCATGTGCCACGGGCAAAGATCGGAAGGGTTACGCCAGTACCAGCGCAGCCGTTTTTCCATTTCAGGCGCATAACGCTGAACCCAGCGGTAAATCGTGGAGTGATCGACATTCACTCCGCGTTCAGCCAGCATCTCCTGCAGCTCACGGTAACTGATGCCGTATTTGCAGTACCAGCGTACGGCCCACAGAATGATGTCACGCTGAAAATGCCGGCCTTTGAATGGGTTCATGTGCAGCTCCATCAGCAAAAGGGGATGATAAGTTTATCACCACCGACTATTTGCAACAGTGCCGCCAAGACCAATGTCGCTCCCGATGCGGTAGACTTTGCCGGCCACGGCGTCCAGCACCGGCGCGAGTCGGGCAAAGGCGGCATCGCTCCCGGAGGCCATCACCGTCATGTCGCCGGCGGCCGCTTTCACGGCGCCGCCCGATACCGGCGCGTCGAGCATCAATAGCTGGTACTCCGCCAGCGCCTCGGCAATGGCCTGAGCATCGGCGGAGGCGATGGTGGACGACACCATCACGACGGTGCCCGGCTTCAGATGGGCGGCGAGGCCGCTCTCGCCGAACAGGATCCCCCGCACCTGGGCGGCATTGACCACCAGCAGCACAACTGCATCCAGTTCCGCGGCGAACGGCACCGCGCTGGGGCCCGCGCCTTTGGCGCCCGCCGCCAGCAGTGCGCGACAGTTGTCGGGATTGATGTCAACGCCCCAGGTGTTCAGGCCCGCCTGCAGGCAGGCGCGGGCGGCGCCCATGCCCATTGAACCCAGTCCAATCACGCAGACGTTAGTGTGTGCAGCCATGCTGGTCTCCTTGTGAACATTAGTTAATTAATGTGATTTAATGATAGAATAAAGCGTTATCATGTGAATTTATGTGAGAGTTATCACGAATAACCCACATGAATATAAAAAATTCACAGGCGGAAAGAGGCTGCACAGCGGCGATTCTTGCCCTAAAATAGCGTAAAAAACAGCGGCAAAGCGCGATAACAACACCGGGTGTCGCCAGGGCCGTAGCCTGGGTGGTGACGCCTGGTGTGAAAAAATGGTAAGGGGAGAGCGGTGATTCCAATAGAACGACATCAGCGTATTTTAGCCCTGGTGGAGCAGCGTGGGGCGGTAAGCATTAACGAGCTGACGGAGATCCTCGGCGTGTCCCATATGACCATCCGTCGGGACGTCAGTAAACTGGAGGAGCAGGGGCTGCTGGTCAGCGTCTCGGGCGGCGTACGCGCCGTCAGCCGGCTGGCCGCGGAACCCAGTCATCTGGTGAAAAGCACGCTGCAGAGTGAGGAGAAACAGGCGATCGGCGCGCTGGCGGCGAGTCATATCGCTAAAAACAGTTGCATCTATCTGGATGCCGGGACTACCACCCTGGCGCTGGCGCGGGCGATCCTCGACCGGAACGATCTCCAGGTGGTCACCAATGATTTTGAGATAACCCAGCTGCTGATCGATGCCAGCCAGTGCGGCGTGATCCACACCGGCGGCACTCTGTGCCGGGAGAACCGCTCCTGCGTGGGCGAATCGGCGGCGCGTACGTTACGCCACCTGGCTATCGATACGGCCTTTATCTCCGCCAGCGGCTGGGACAGTCGCGGGATATTCACCCCTGATGAGAACAAGGTTACCGTCAAGGAGACCGTCAGCCAGGTCAGCGCGAGAAGCATCCTGCTGTGCGACAGTTCGAAATACAATCAGGTGGCCACGTTTATGGCGTTACCTTTGACCCGGTTCACCACCATCATTACCGACCGGCATCTTTCCGATGCCGCCGCCAGTCATATCGCCCGGCACGCCTGCGAGGTGCTGCGGGCCGGATAACGCGCGCGGCCACCGCCGGCTTAGCGTTGCCAGTGCTCGATCAGCGCCGCGCCGATCCCGGCGATTTGCTGATTTCGCTCGGCCATGCTCGCCGGCGTATCCCGCAGATAAATCACCACAATGCGCTCTGCTTTGTTATTCGGGCCAAGCAGGGCGACAATCCCGCGCGCACCCCGCTTGCTAGCTCCGGTCTTATCGGCGATAAACCAGCCCGCCGGCAGCACGGAGCGGATCAACGGTCCGGCGACCCGATCGTCCACCATCCACTGCAGCAGCTGCCGTTGCGAACGGGCGCTCAGACGCTGGCTGGTCAGCAGCTTGCGCAGGGTCGCGGCCATGCTGGCCGGGGTAGTGGTGTCGCGGGCGTCGCCGGGAAGCGCCTCATTCAGTTCCGTTTCCCAGCGGTCAAGGCGGGTGACGTTGTCGCCGATCTGGCGCAAAAAGGCAGTCAATCCTGCGGGGCCGCCGACGGTGGCCAGCAGCAGATTGGCGGCGCTGTTATCGCTCATGGTAATGGCGGCGGCGCAGAGTTCGCCGACCGTCATGCCGTCGGCAAGGTGTTTTTCGCTGACCGGCGAGTAGTCCACCAGATCCTGCTGGCGATAGTGGATCTTTCGCTCCAGCTGTTCGTCACCGGCATCCACCCGCGCCAGCACTGCGCCGCAGAGCACTACTTTAAAGGTGCTCATCATGGGAAAGCGTTCATCGGCGCGCCAGGCGGTCAGCGTGCGGCCGCTGGCCAGATCCATTTCTATCATGCCTACGCGGCCCGACAGCTGGCTTTCGCTTTGTTTAATTTGCTCAAGCGGCTGCGGGCTGGCGTGTACCGCCAGCGGCAGGGTGGCTAACAGGGAGATAATACACAGGCGAATATAACGCATAACCACAATACATCCTTGAGTGAAGGCCGATAAAGGCGAGTAAAGAAGCGACAAATAAGAATAACCCGGCGTTGGCACTGTTGCAAATAGTCGGTGGTGATAAACTTATCATCCCCTTTTGCTGATGGAGCTGCACATGAACCCATTCAAAGGCCGGCATTTTCAGCGTGACATCATTCTGTGGGCCGTACGCTGGTACTGCAAATACGGCATCAGTTACCGTGAGCTGCAGGAGATGCTGGCTGAACGCGGAGTGAATGTCGATCACTCCACGATTTACCGCTGGGTTCAGCGTTATGCGCCTGAAATGGAAAAACGGCTGCGCTGGTACTGGCGTAACCCTTCCGATCTTTGCCCGTGGCACATGGATGAAACCTACGTGAAGGTCAATGGCCGCTGGGCGTATCTGTACCGGGCCGTCGACAGCCGGGGCCGCACTGTCGATTTTTATCTCTCCTCCCGTCGTAACAGCAAAGCTGCATACCGGTTTCTGGGTAAAATCCTCAACAACGTGAAGAAGTGGCAGATCCCGCGATTCATCAACACGGATAAAGCGCCCGCCTATGGTCGCGCGCTTGCTCTGCTCAAACGCGAAGGCCGGTGCCCGTCTGACGTTGAACACCGACAGATTAAGTACCGGAACAACGTGATTGAATGCGATCATGGCAAACTGAAACGGATAATCGGCGCCACGCTGGGATTTAAATCCATGAAGACGGCTTACGCCACCATCAAAGGTATTGAGGTGATGCGTGCACTACGCAAAGGCCAGGCCTCAGCATTTTATTATGGTGATCCCCTGGGCGAAATGCGCCTGGTAAGCAGAGTTTTTGAAATGTAAGGCCTTTGAATAAGACAAAAGGCTGCCTCATCGCTAACTTTGCAACAGTGCCACAATGTGCGTAATGACCCCCCTGACCTCCAACCCGTCCTCATCGTCATCAGGTATAGCCCTGAAGTTATCCAGCTTATCGAGTTCCTGGAGACACCGCTTGGGGTGTAGTCGCAATCGCATGATCCTGAATTCTTGTTCCAGGACACAGAGCACCAAAGACCCGTCAACGGGCGTGCCCCCAAAATCTACTATGAGCAGCGCACCAGGCTTTATCCCTTCCCGGAAAGAGTAGGTATCACTTCGAAAAAAGAATACTCCCGGCGCTCCTGTATTACAGTGCTGATCGAGTGACAGCCTGTTTTCCTGGTAGTCGGTTGCTGGCGATGGAAAACCCATTTTTAACGGCCTCCGTTGGGGTTAAAAAGCATGAATGTCCGGTTCTCTCCTTCCTGAGTGGAAATGTCTTTAAAAACGGTCACGTAGGTTTCAATCCACTGATTTGCTTGGCGACAACTCCAGTGAAAATTAATCAGCGCAAGCTGGCTGACAAAGGCTTCTGTCGTAACTGTCTGACGACCGTTCGGCTCACGCTTAATGCTCGCACGCCAGGCTGAGTCAATTTCATAGTGTCTAGGCATGAAAAACCTCCGATCAATATAGCTGTATGCATATGGGTCTTCCCTTGTTGTGGTGGCTGAAGGCATGATAATGGTGTATTTAATCGCCAGAGGTCACCGCCATGGACGAAAAGTCCCTCTACGCTCATATTCTCAACCTGTCCGATCCGTGGCAGGTAAAGTCCCTTTCTCTCGATGAAAATGCCGGTTCTGTTACTGTCACTATTGAGATCGCTGAAAACACCCGGCTAGCCTGTCCGACCTGCGGTAAATCCTGTTCTGTTCACGATCACCGTCATCGTAAATGGCGCCATCTTGATACCTGCCAGTTCACCACTATTGTTGAAGCCGATGTTCCACGAATTATGTGTCCGGAGCATGGCTGCCTGACGTTGCCTGTTCCGTGGGCTGGCCCCGGAAGCCGGTATACGTTGCTATTCGAATCGTTCGTTCTCTCATGGCTGAAAATCAGCACCGTTGATGCTGTCAGGAAGCAACTTAAGCTCAGTTGGAATGCGGTTGACGGCATTATGACCCGGGCAGTTAAGCGAGGTCTTGCCCGGATAAAAAAGCCATTATCCGCCCGTCATATGAATGTGGATGAGGTCGCCTTTAAAAAAGGACATCGTTACATAACGGTGATCTCCGATCGCGATGGTCGGGCGCTGGCCTTAACGGATGATCGCGGCACAGAGAGTCTTGCCGGCTATCTTCGCACGCTCACTGATGGGCAGTTGCTGGCTATCAAAACGCTCTCAATGGACATGAACGCGGGCTATATAAGAGCAGCGCGTATCCACTTACCCAGTGCGGTTGAGAAAATCGCCTTTGACCGCTTCCATGTGGCGAAGCAACTGGGCGAGGTAGTTGATAAAACCCGTCAGAATGAACATCCGCACCTCCCTGTTGAAAGCCGACACCAGGCAAAAGGAACCCGCTTCCTGTGGCAGTACAGCGATAAGTGGATGACCGAATCCCGGCAGGAAAAGCTGATGTGGCTGCGTGCACAGATGAAGCTGACGAGCCAGTGCTGGGCGCTGAAAGAGCTGGCAAAGGATATCTGGAACAGGCCATGGAGCGAGGAAAGACGGAGTGACTGGCAGAGATGGTTGGCGCTGGCGGCTAACAGTGACGTTCCCATGATGAAAAATGCCGCGAAAACGATAGGAAAAAGGCTGTACGGGATCCTGAATGCGATGCGACACAGTGTCTCAAACGGAAATGCGGAGGCACTTAACAGCAAGATCAGGCTGCTGAGGATAAAAGCCAGGGGATACCGAAACCGGGAGCGCTTTAAACTGGGGGTGATGTTCCACTACGGAAAGCTGAATATGGCGTTCTGAGCCTTCCCACCATGATCGGGGAAGACCCATGCATATACAGTATAAAGAATCAGTTTTATTCTTCAACACACTGTTTATTATGTTGTAACTACTCCCTACCTTCAGGTTCATGGTTCATTTTGTTAACGGTCTGTTGCTTCACAGCTTTGCTGATAGTCGCCCGGCTACAACCCAGCACTTTTTGAATCTGGCTCCAGGAGCTGCCGCTCGCAATCAGCCTGTTTATGGCGTCATAGCGAGACTGATTAACCTGGCGGCCTTTATACTTCCCTTCCCTTTTCGCCCTGGCGATCCCCTGCTGCTGCCGTTCCCGGCGCTGTTCATAGTCTCGCCTGGCGACGGCGGCCAGCATATCCAGCAACATATCGTTTATGGCCGAAAACATTCGGCTGTCAAAATCATTGTGACCCGATGCGAGCCAGGTTGTCGGAACGTTGACGGCCACAACCCGGATATCTTTCTGCCGGATCATTTTCTTCAGTGTGTTCCAGTCCTCCCCTACCAGGCGTGAAAGCCTGTCCACATCCTCTACCAGCAAGATATCGTTTTGCTGACAATCTTTCAGGAGCCGGAACAACTCAGGGCGTTCAAGCTTAGAACCTGATTCATTTTCAATGTAGTAGTTACAGATGCTCAGGCCGCGCTCGATGGCGAAGGTATTTATCGTGTCCAGGGCGCGTGTTGCATCCTGTTCAGTCGTGGATGCGCGTAAGTAAGCTCTGACAAAGCCTTTCGGTACTGTTTGAGTCATTATAGGGAACCAGTTCATTTTGAGTTAACCAGACACACCCATACAGTACCCATTTCAGTTGGTTCAGGGAAGGCACACTCAAAAAGAACCAGCAAACAGACCCCAGCCCTATAGTGACAACGTCACACTAAAAATTTACAACATAAAGATGATGTAAAGACACACCAAGATTTACACCATAAAGCGCACATAAAGACACAGCAACATTTACCATGTAAAGACACCATAAAGATAAATATATCTTTATTTGTGCTTTACATGGTAAAGCCATTGCATTATAAAGACACTGTAAAGACAAAAGTATCTTTACGAAAAACACACCATGTAAAGACATTCATATCTTTACATGAGCTTTACAACATAAAGAGAGGGATTCTGAAATGGGTAAAATCTTGCTTGTCGTATCAGATAAAGGTGGGGTAGGCAAAAGCACCTACGTGGCTAACACAGGCTCAATGCTGGTCAACAAAGGTAAGTCGGTAATTATCCTGAAGACAGATAAAAACCACGATCTGTTGAGCTGGAACGAAAAGCGGACAGATAACGGCTTACTCACTATCCCGGTTCACGCAGCTTACGGAAACGTCAGCAACGAAATTAAGCGCCTTAGTAAGCTGTGTGAAGTTCTCATTGTCGATTGCCCCGGCCACGACAGCCAGGAGTTCCGTAGCGCCCTGACAGTATCAGATATCGTGGTGACACTGGTTAAGCCGTCCTCTGATTTCGAAAGCGAAACACTGACCAGTGTTACAGAGAAAATCCGCACCGCTCAGAAAGCTAACGCAGCTCTGGAACCGTGGGTGCTGTTCACCAGAATCAACTCAGCGAAACCCCGCCACAGAAAAGCGGCCATTGATCTGGATAAATTACTGCGTTCGGACAACATCTGGATTCAACCTCTGAAAACCCGGATATCCGAGCTGGATGTGTATGAAAGCGCCTGTAATGAAGGGGCAGGGGTACATGACGTTAGCCGGGCATCGAGCCTTTCAACAGCGAAAGCACAGATTGAACTTGTGGCACAGGAAATTGGCATTTTATAGAAATATCAACAATATTCATCGAAATCTTTACATTGTAAATACACCATAAAGATACTATGATATTTACAATTAATGCATACCGTACAAGGGGAAATTTTCATGGCTTTAAAACTAAACCGCCCGAACATCGATGAATCTCAGCAACCAGCAACAAACGCAGAAACCGCGCGTTTTATCTCCGGCGCTACCAGAGCGCCTGTAGCGGGTAAACCAAAGTTGATGAACTTCAGACTGCCAGCGTACTTTGAGGAGATCCTGGAAGCGGAGGCGCAGAGAACAGGCCAGAGCAAGACAACCGTTCTAAAGGCAGCGCTGGCCGCATTCGATAACCTGGACGAGAACCTGAAAAACCACTGGCTGCTTGAATCTGCAAAATTAGGGTAGCGTTATGGCTAAGAAAAAGAGATATGTTGTGATGGTTAGAGATAAACCAAATACAGCGGCAATCAGCGATTATTAGCCTGGTTAGTATGGTTCGCTAACAGGCATAATAAGGCCGTTGCCTATGACTGCGGAGAATGGATTGTCGAACCATCACATTGGTTAAGAATTGGAAATCCAAAATAGAAAGAAACCTAAGGAAAGAAAAATGTTCATATTCAGATATATTAAAACTCTTATTAATATCTACTTGTTATACATCCAACCGATATTGGGGATGTTCCTTATAGGTATAGGCTTATGGGGGTTCTATGCACCTGATGAGGGAACTTGTACTAATCTCAATATTGAGTTATGCGCTGTATCAGCTATTGATGTTCTTAAGCCTGCTATTTTTGTATTTTTAGGCATTATTACCGTTGCCGGATGGTACTTTACCGAACTTCAGCCGAAGCTAAGGGGGGAGGGCAAGTATATGAAGGTAAAAACAGGTGTAAAACCGGAAGATTTCGTTAAGTAAAAACCGAGCCTAAAGACGTTAACCGTGGGGGATTTATGAATAAATCATGGCTTGAACAACAGGTGCAAAATGAAAAATTAAAAAAAGTACAGGAAGAGAAAAAAGATGGCACTATCCAAAAAGGATTGTACAGGGTTCGTATAAAAGACAAATACAGGTGGGATTTGTGGGAAAATGATTTCAGAGTTGTTATTGCTTATTCGAAAGATGATGCAGAGCTAAAATTTACAGAAATGACAGGTTTTAATGAGCATTACTATATAGAAAGCATTGAAAAAATAGTGCTTTCAGATATAGAAAAAATCTTACTGGAAAACTGAAAAGATAGTTATCCAGAGGTTTAGTTAAATAATGGCGTCAGTTTTTCAACCAGTAGCAGATCGCTTAAACCGTCTAAAGAAACAACTTCTATACCAGAATCAACAAGTGAATCAACTCCACATACTTCACCGAAGCCGATAACCATAATCGGCTTTCGGTCTATCAAATCAAAAATCACCAAAGGCAATATTACACTGTTAATCGCTTCAAACTCCTGAAAAAACATTTCAGAGCCGTATTCGCTGGCAGGAAAAAGAATCTCACTTAATCTCAAACCATAAAAATATGGTAGTGTTCTTTAAGGTTATCCTTTAAAATATTAACAGCCTTAGAATATTTTTCGTTAATAAACTCTTTAACCTGATATTTCATAACTCTCTCCAACAAGAACCGACTGTAGGTCACCGGGCAAACGTTGCGGAATGGCGTCAGAGACGTCATTTTGCGGCGTTTGCCCTATCCTGCATCGCAGTGAAAAGAACTCGCCTCGTGCGCTATGATTAACCGATGATAAAAGGCTGAAAGAAGCACTGCAAGCCGACTGGATAACATTGTTGCTTCACGGCTGATCATCATTGGTTGCCTCAGTGCTAGTGTGCTTTGCATCAGGAACCAGAAGACGATACCACGGCATTTTTTTGTTAAGCAGCTCCATCAGGGGAGTGGCAAACAAACTCAATCCCCACAGCGTTACGATTACTGGAATCGTGAAATTTTGCGGCGTCCAGAACTGGCGCTCAAGGTGGAACCATGTAACAACACAGAGCATTCCACCAATCCAGAAAATAGCTTTACTGAAAGCCCCCAGGACTGCCAGAGAAATAACAGAGACTAAATGCAGTGCGCCAGCCGTAGCAAGACGTAGATAAAACCAGAACCATTTAGCGTGCCGGATAACCGGGTTACCGTTCGTTTCTCGCAATCGTTTTTTACGTTCCCGGATGATTTCTTTTTTCAGCTTACTTCGTTCGGCTCCCTGCGGAAACTCATATATTTTAGCCATGTTTTGCCTCACTTATTCTTTTTTGGTGGAACGACTTTGTCAAACTCCTCTAAAAATGCCATATCATCTTCCGAAAAGGATGAGGATTTTTCATTTATACTAAACTCAAATCTAAGCATGTGAGCCATTCTCCCTTTTTTCCCAACAACAGAGAAAGATAACTGATTTATTTCCGTTTTCTCAATTATCTCATTAATGCTTTTATTGATAACATCACGAACAAAAAATGAGTATTTAGGATACTTATATTTCTTCTCCCCTTCCTCAATAGTATAAAGTCCCATTTCCTCTTTTAAATCATCCACGGATATATCAAAAGATTTTTTTGAACTATTCTGACTGTAATATTTCCTAATGACTTGATAAAGGTTTGTTGAGTTTACACTGTTCAACAACACAACTGATTTCAATACCTGAGTGGTATATCTATTTTCAGAACCAATCAACTTAGAAAAATATCGCTTAGCTGTATTTGTGAATTTTATCCGTACAGTTGCAAGATGATCGTAATAGGCACAGAATTCTGTTAAGTTTAAATCCAAGCGATCAGGTATTTTATTCTTAGATGATAGTATCCCAAGTTCATCAGCCAAATTTTTTAAATCATCTCTGTTTAAAACTAACGTAGTTGAAGAAAGAACTTTAGCTCCCTCTTTCAGAGCTGCATAAGAGGCATATTTATCTAAAGAAGTAGCTTTTGAAAAAAAGTCAGCATCAACCTCCAAAATATCATCATCTAACATTTCCTTTGTATCAATCTGGCAAAGAGCCAGAAAAAGCACTCTTTTTGCTGAAAGGGAAAGGGATGCAAGAGTTACCCCAACTTCATTTCTATGTCTAACTTTCGTTAATCCCGTTACTGTCTTCACGATAAGCCTCATATAACCTTTCCTATGTAGGCAAATCATAACCCCACAAAGGTTGTAATTCAAGACAACAATACAACCTTTAATTAAAAAACCTCTCACCTTTAGATATGAAGATCTGAGGATCTTCATATCTAAAGATAGCAACAACCGCATGTTTCACTTGATGTTCAGTCAAACTGTGGAAACCGTTAAAAATCTTCTCACCTTACGTGAAAAATCCTCTCATCTTTAGTGAAAAATCTAACTACCTTAACTTAAAAATCTTCTCACCTTACGTGAAAAATCCTCTCACCTTACGTGAAAAATCCTCTCACCTTTAGCAATTTTTTTCCATAATTTTCAGGTAATTGAAGCGAGCTAAGTTTTTAAGTTTTTAAGATCAAGAAGAAAATATAAGGCTTTTAATGCACAGCGTCTGTGGATAACTCAGCATAAATCCAGAGGATAGATCCGTCTGGCCTCCCTGAGCACTGCACCACCAAAAATTGAGCAAATATTAACCTTAGCGCGTAAGTGCTCTATTGAAACCCCGTGGAAATTCCGCTTCCTCGCTCACTCGCGGGCTTCGCCCTGTCGTTCGGCTGCGGCGAGCGGTGTAATCTCTCTAAAAGATCCAGACGGGATCCTTTTCCGATCCAATTCTTAAATGCTCTCAAAAGCCCTCAGAGGCGTTTTAACCGCTTTAACCCGGTCATGGTTCAACGGAGCGGGTAAAATGCGCTTAGAACGTCATACAGCTCGTTTTTAACCTGATCCCTAATCAGGGGGTATAACAGAGGCGGCCAAAAAATCCGGCCTTGCTCGTCAGAGTATCTACCGCGCATTATCCCCGAACGGAAACCCGACCATCACAACACTTGCACAGCTTGCAGCAGTAGCCGGGTTACAGTTCACTCTCAGTAAGCCCAACCACTAACCTTTCAGACAAGCCCCTCATACCGGGGGGATTATTTCTTATGCCAGCAAAATTTCACGACCGGCGAGGATGGCCAAGTGTTTTTTGCTGGCAATTTTCGTGCAGCAGCTGGCGCCCGGATTCATTTTTTGCTGTCAGGCATCAATCGGTTTCAGCAACCCGCCCCGTAATTCGCTGCTAACGCATCTCATAACGCGCAGATACAGCGCCCCGCGCCAGTGTTTCACACTGACCCGGCGTACCGTTCGCAAGCTCACGCGCTAACTGCGACAGGCTAAACATCGTCTGATAGTCAGAAATTGGCCTGGCAGGGGTGAGGGGGGTTATCGCTCTCATTCGGGCTTGTTGGTTCGCCAGTCCTGGCGAACGTCTCCGGCGCGATGTTACGACAGCGCATTATGTAACTACAGTTCGCGCCTCTTTCGGAAATCCCTTCATGCGCGGTAAAGCAATATACATCTTTATGTAATCTTTACAATGTAAAGCATTAAAAATGATTCCATAACCATCATTTGATGGTATAATGAATTTGTTGGTTGGGAATGGTTCCCTGCCAGATTCCAGAAGCCCACACGGGCGGGAGCGAAAAATGAATATTCAGGAAGCATTAAACGTTTTTGGTTTATCCGGTGATCTGACTGAAAAGGACATCAAGGCGGCATACAAAAAAGCGGCTTTAAAATATCATCCAGACCGCAATCCATTGGGTGCCGAGCTGATGAAAGCAGTAAATGCTGCGTTTGATTTTCTCATGGCTAACATTGATAAAATCAATCAGTTTCAAAGCACTGATGAAAACGCACGTTACAACTACGGTGAAGATCTGGAAAAAGTTCTGAATACGCTTTCCGGCCTTACAGGGATTGTCTATGAAGTTATTGGTAATTGGGTCTGGATTAGCGGAGAAACTAAAGAACACAAAGACATTTTAAAGGAAATGGGCTGTAAGTGGGCATCTAAGAAAAAACAATGGTTTTACCGTCCTGAAGAACACAAAAGCCGCTGGAACCGTAAAGAACACAGCATTGAAGAAATTCGTGAAATGTATGGTACAGCGGGTAAACGTAAGGCGTCAGGCTGGACACGTGTAGAAGCAAGCGCATAACCGGAATGGGGGCGAAAGCCCCCTAGAACATGGCAAAGATTGATTCGGAGACACGTTGATGAAAAACAAGACCATAACGGAAGCGGAGCTGATAAACATTTTTGAAAGTTACGGAGCCTACATTTGCCCGGATGAGATCGAAGTTACAGCCAAAGAATGCAATGAAAATGGTTCAGTCTTGCACCGTGGCTTAAACGCAGAAGGATGGGCGCATCTTTTTGCGAAGGAAGAAGCATATCAGCAGGAATGCGAAGCCCAGGAAGCTGCAAGTGATGATGGACACTTTGATGAATAGTCTTAAAACTGCCGTAATTCGATTTACGGCTTTTTTATTGAGTTCAAAAGGCTGAATCGATGAAAGAACAGAAAAATTTCTTTGAACGGTATAAACCGGTGTTTGAAATCGTTTGTCGAATCCTGGGGAACGGCTGGCGTGTAAATCTGCTCGATGATTGCCAGTACAGGATTAAGCTAACCTCACCTGATTTTAAAAATTACTCTATACATATTCGAATGGAGAAAGGAAGGTTAGTTATCATTGGAAGCGTAGATAGTCGCAGCTGGAGAAGTCCTTACCATACTTGCACAGTATCGCCAGAGCGTAACCCTGTAGAAATCGCCGCCGATATTGAAAAGAAAATCCTGTCGGATGCTTTAGATAATGTAGATATGGCGCGAGAGTATGAACAACAGTTACAGCAAAAACGCGAGAAAAAACTGATCCTGAAAGGAATGCTATCTCGTTTAGTGCATCTTGAAAGCTGGCATGGGACATTGACCGGGTTTAAAGTTGAGAATGGGTTAGATGGTAACGTATCAGAACGGGGCGACGGATATGAAATGGTAATTCGTGGCCTGAGTGTAGATCAGCTCATAAAGGTAGCGGGGTTTATTAAACAGCTATGAGGCAAAAAATGAATGAAGTCCTGAGCGACAGGAAAAACAAAGGTAATGTAACATACAGGATTGTTGTGTCTGAAGATGCAACACGTTTGTTTATTGAACTTGAAAAACTGATGAAGGTACGCTCTAAAGAAGCGGATAAAAAAACAACTAAAAAGATTGTTTTCCAAAAATCATTTTATTACGAAGAATTCTCGAACGTTAAAGATGAAATTGATGATCCAATTTTATTGAAATTCTATACAGATACTATTGTGAAGGTACAAAATCATGAGTTTTAAGCCAACACCTGAAGAGGTGAAACAAGCACGTATTAAAGCTGGCTTTACGCAGCAGGAAGCCGCTGAACGGTTTGGTTTTACGTTATCAGCCTGGCAAGCCAAAGAAACATCCGGTAAAACGTCTCGTGGTTTAGCCGCTGGTACGTATGAATTACTTTTGCTCCTGGCTGATGAACATCCAGATTACCAGCTCGTTAAGAGAGAAAAAAATCAGGATAAAGTGACTAAATAAATTGCATTACCATCATATGATGGTATAATTTATTTGTAGGTCGGGAATAGTTCCTTACCATGATCCAGTAGCCCAAACGGGCGGGAGACTAAAATGAACACGTATGTTATCACTTTCCAGGCTTTAAACACTAAGAAAGAAAAAATCACCTGCTCTGCTTTTGTCCATTCAGAAACTCTCTTCCTTGCCGTTCATTCTTTCGAAGATAAAAATCGCGGTCGTGGTTATGTTATCACGTCTGTTAGTGAATTGTTGTCCGAGGAACTGACGGCGAAAAATTCCCTCAAGAAAAATATTAACTTTTGGTTTAATGAGTGTGGTTTAAGTAAATCTGAGGTTATAAACGAAGTAATTAACTGGAAGAATTTCGCATATACCCTTAAAGAACTGGAGGAAGCCAAAAATGAAGCGATAAGAGAACTGCGGAGTTAATCAATTAGGGGTGGTATCAGGTTGTGGAGGCAACCTGATACCGTGACACTCAATTCCCTCGCGAGGAATATAAAATGTCGGAACGCATGTTATCAGCAATACAGACTGTTGAAAAGGGTGGGCGTCCGGTTTTTCCCTTGATGCCATTCTCTGCTTTTCCTGAGTACATGGCATTACTCAGAAAAGCCCTGGAAAAGAAAGAAACAAAAGCACTGATAGAAAAACAGGAGGTGCTATGAAAAAACAGGAGTTTTTAGATTTTATCAGTGCAGAACAGAGGCGGGGAGCTGTCCGGTTTTCGCTGGGATTTAACAGCAAAGGGGAAATTGTATTGCACTGGACTAACGAGGCCGGGTTAAGAGTCTGGAGTATACTAAGCGGTAACAGGGGAAAAAGTCCCAGCCGGGCAAACCGGGAAAGAATGAGTAACCTCCGCCGCTGGCTCCATGATGCCCGGCAGGGCATGGAAGGCGACACACCAGAGGCAGAATAAAATTCCCGTTCCTGAATCGCCGAAGCCCTCCCGTTTACGGGAGGTGCTTTTTTTGTATATTCCGTAAAACAGCGCTCGCGCCCCCTGCCAGGCCATTCGCGTAGCGAATTTATTTCCGCCCAAGTTAGCCGCGAATCCGTTGTCTCCGGTCGCAGACGCACCAGATCACAGCCACGATAGCCTTTCATTTTTTGCATTTCGATGGACGCTGAAGCACAATTTATGTGTGGAGGTAGTTCACTGCGATGCAGGATAGGGCAAACGCCGCAAAATGACGTCTCTGACGCCATTCCGCAACGTTTGCCCGGTGACCTACAGTCGGTTCTTGTTGGAGAGTTTTATGAAAAAAGTTCAATTCAGGATTGAAGAAAGCCAACATGATGATCTACTTGATTGTCTTAAAACACTTTATCCAGATGAACCGGGTTTAACCGTGGCAAAAGGAATGAAGCTGCTTGCCAATGCTTTACTGAAAAGCAAGGTTAAAAATGAAGATGTTAATGTGTCAAAAGACAATGATGATTTTATCAAAACCACAATGTATTTGACAGGGAAACAAAGAACTTTAATAGAGAAAGCGGCTCATCGTCATGGCTGGAATTTATCCAGAGAATGTCGTTATCGAATCCAGACGACACTTGAAAATGAACTTGATTTTTTCGATCAGGAACTTCTGGTGATGAATCGTTGCCGTAATGCAATAGATAAAATAGGGCGAAACTTTCATTATATTATCGTTAATGATAATGCCAGAGTACTTGATAAAGATGGGTTCTATCAGGACGCTGAGCGGTTGAGTTCAGAGATAATGAGTCTCAAAAGTGAGTTTGAAAACTACATTATGTTATGCAAAGGGCGAACTGTTTCTAACAAGGTTGAGGTGTAATCATGGGCGTTTATGTTGACAAGGAATTCAGGGTTAAGCGTAAGTCCTCTGAAGCTGGCCGCAAGTCTGCTTTCGCCCATAAAGTTAAAAATGGTGGTAAAAACTATCAACGCAACGTTCAGGAACGTATCAACCGCAAGGGTGCCAGTAAGGAGGTTGTTGTCAAAATCTCAGGAGGTGCGATTACCCGGCAGGGAGTTCGAAACAGTATTGACTATATGAGCCGGGAGTCAGAGCTGCCAGTGATGAGTGAAAGCGGTCAGGTATGGAAAGGTGATGAGATTCAGGAGGCTAAAGAGCACATGATAGATCGTGCTAATGACCCTCAAAATGTTTTCGATGATAAAGGTAAGGAAAATAAAAAAGTAACCCAAAATATTGTGTTTTCACCGCCTGTATCAGCAAAGGTAAAACCTGAAGATCTGCTTGAGTCTGTCAGGAAAACGATGAACAAGAAGTACCCAAATCATCGTTTTGTGCTTGGCTATCATAACGACAAAAAAGAACATCCACACGTGCATGTCGTTTTTCGTATTCGTGATAATGACGGTAAACGAGCTGATATCAGGAAAAAAGATTTACGGGAAATTCGTACAGGTTTTTGTGAAGAGCTGAAGATGAAGGGCTATGACGTTAAAGCCACTCATAAGCAGCAGCATGGACTTAACCAGTCTATTAAAGATGCGCACAAAACAGCGCCAAAACGACAGAAGGGCGTTTATGAGGTTGTTGATGTTGGCTACGACCATTATCAGAACGATAAAACCAAACCAAAGCAGCATTTCATAAAGCTTAAGACACTGAACAAGGGCGTGGAGAAAACATATTGGGGGGCTGATTTTGGTGAGTTAACCACTCGCGAGAACGTTAAGAAAGGTGATCTTGTTAAGCTGAAGAAGTTAGGACAAAAGGAGGTGAAAATCCCTGCACTTGATAAGAACGGTGTACAGCATGGCTGGAAGACTGCACACAGGAATGAGTGGCAGCTAGAGAACCTGGGGGTTAAGGGCATAGACAGAATCTCTTCATCCAGTAAAGAGCTGGTGCTGAACAGCGCGGAGATGATTAAGAAACAGCAACTCCAGATGAGAAATTTCTCGCAAATAAAACAGTCGATGATTCAAAGCGAACAGAAAGTGAAAATCGGAATTCGTTTAGGATAATTCATACTCTCCGGTAACTTATTGATTAAGATCGATTTATTTATCTTGTTAGCAGAGTTTTTGTAAAAAGTAACTAGCGGTAAAAAATTTAGTTAACTTGACTATAAAATAAGCGCATGGTGATCAATCGATCTGCTTTTTTGTTATCAAATAGCATATGTATCTAGATATAAATAAAATACAAGCACAAACAAAATGAAATGTGTCGTAAATGCGATCTAAAGATCTGCTTGTGTGGTTGAACGCAGCACATGTAGCTGATAAAGTTTTTATGAGATGTAAATTTGTCCTTTTGTTTTTTTTCTCTGATTCGGGGTCATCCGAGTCGAGAGAGCGCCTGAAAGGAAAAACTATGGAATGGTATGTTTTGCAATTCACCACTACGAGATTTGCAGCAGTTTTTGCTCATCTCGAACGCCTGAACTTCTCTTACTTCTGTCCTATGGAGACTGAAAGGTATCGTCGCCCGGATAAGATAATTTCATATCGAGAAAGACGTTTGCCGCTTTTTCCCGGTTATCTTTTTATCCAGGCAGATTTTGAAGAGGTTCATTCCACTACAATAACTGCTATCCCTTACGTACAACGCTTTATCTCATTTGGCGGTGAGCCTTTACCTGTACCAGAAGATGTGATGGCGGAGCTTTTGTACAGACAATCACATACAACAGCTCAGGCAAATCTTTTAAGGAAATCAATTCCACACGATTTTGCTGAAATTCTACTCATGGATAATCCGCAGCAAAGAAGTATGGCTTTCATCCACTACATCACAGAGAGAAGCTTAACCCACAAAATGAAACGGAAGAAAAATGACTGCTACCCGAAAAAAAACCGCAACGAGACGCAAGCCCCAACGTAATACACTCTACCTGCCCTCAGCTTCTCGTGAGGAACTGGATAAAATTGCTCTGGAGATTGGCTATCGCAGGGGGCGACGTATTTCAAGCTCTGGTGTTGTCCAGTACCTCATAAAAAAATATTCCAGCCAGGCTATTGAGGAGCTGATAAACGAAGACGATGAAGACGATGACGACTTCGATGACTAACCATTCAGTCTAAACTTTCCGCGCTGTTCTGGCCGGGAGAAAATCATGTTATCTACTACTGCTTTTGCGGCGCTTGCTTTGCAATGCGCGGCCAGCGTTCATCCCGATACTGCGCATGAAGTTGCAAGGGTTGAATCTGGTTTTAACCCATATGCTATTGCCGAGATAATACCGAAGGTTGAACGTAAGCCCGGTGACAAAGGCGTAGTGTCCTACTTTCCCAAAACTAAGGAGGCTGCACTCCAGATCGTAAATCAAATTGAATCACGAAATCATCGTTACTCAGTCGGTCTTATGCAAATAACGAGTACGAATTTTGCAAACTTCAATACAACCGCTGAGAAAATGTTTGACCCCTGCGAAAATCTCAAGGTTTCAGAGCAAATTTTGGTGGATTGTTATAAACGTGGTGGCGATATTTTACGTGGCCTGAGTTGCTATTATTCCGGCAATCCAGAAACAGGAACTAAACCTGAATCAGATTTTAATAACACCAGTTATATACAGCGCATAGGGTTTAATCCGCCTGATAACAAAAAAAACTGGGTGGTTCCTTCAGTTAAAGATGCAATCAGAAAGGAGAATGTAACACAGAGTATCAAACCTAAAGAAGTTACCGTATATCCCCAATATGCCATGCGTGGCACTGTTTTAGATGAAAAGGAAACAAACGATGTTAAAACTCAATAAACGTTATTTAACTCTTTCTGTATTTATGGCTGCATTGATGCTTTGTGTAGCAGAACCCGCCTTTGCTGATGATGTATCCACTAAAACAACTGGCTTCTTGCAGAAGATTATCGATTTTCTGACGGATATCCGTAAGCCAGCAATCACCATCATTGCGCTGGTAATTGGTTATATCGCTATCTTCTCACGCCAGCATGCAGCGTGGATTACGCCTTTAATTATCGGGATTATCATCTTTATTGTTGCGCCATATCTGCCTGACTGGTTAGCGTAATATGTAATAAAAGGGGGCGTAAAAATGAGTACCGTTTTTAAAGGGCTGACACGCCCCGCTTTGATTAGGGGGCTGGGCGTTCCGCTCTACCCCTTTCTTGGAATGTGCGTTATCTGCGTTTTGCTTGGTGTCTGGATTCATGAGGCTATGTATGCCCTCATCCTCCCTGGCTGGTATGCCATCAAGCGAGTTACGAAGATAGATGAACGCTTTTTTGACCTGCTTTATCTGCGAATGCAAATCAAAGGTAATCCTCTGGCAAACAAGCGCTTCAATGCCGTCCATTATGCGGGGAGTTCGTACGACGCAGTCGATATATCGAAAGTGGACAATTTTATGAAGCTTAAAGACCAGTCTTCTCTTGAAGAGTTAATCCCGTATTCATCACACATCACTGATAACCTCATTGTTACCAGAAACCATGATCTACTGGCGACCTGGCAGATTGACGGAGCTTATTTTGAATGTGTAGATGAAGCGGATCTAGCCTTGCTTACTGACCAGCTCAATACGCTGATACGTAGTTTTGATGGGAAACCCGTTACCTTTTATACGCATCGTATTCGGGTTAGAAAAGAAGTGCGACCTGTATTTGACAGCAAGATCCCATTCGTCAACAGAGTGATGAACGATTATTACGAATCGCTTTCGGCGGCTGAGTATTTTGAAAACAAATTATACTTAACCGTATGCTATAAACCGTTCAGCGCTGAAGATAAAGTGACGCATTTTCTTTCAAGGAAAAAGGGTAATAAAAATATCTTCGAAGAACCCATTAATGATATGAATGAAATATGTGGCAGATTGAGCACCTATCTCTCCCGGTTTCATTCCCGTCGTCTTGGGTTATATGAAGAAAATAATATAGTTTATTCAGAACAGCTTACGCTGTTCCAGAAACTGCTATCCGGGCGCTGGCAGAAAGTCAGAGTCACCAATAGTCCGTTTTATACATACCTGGGTGGTAAAGACCTGTTCTTTGGTAACGATGCTGGACAAATCACTGCCTCTGACCATGCCCGGTACTTTCGTTGCATCGAGATCAAGGACTACTTTCAGGAAACGGATGCCGGAATTTTTGATGCACTGATGTATCTCCCGGTTGAGTATGTACAGACTTCCTCTTTAACTCCGATAGACAAGCAGTCTGCAATCAAGGCGCTGGATGATCAGATCGACAAACTCGAAATGACCGATGATGCCGCTAAATCCTTGCTCGCTGATTTGAAAGTTGGTCTCGATATGGTCTCCAGCGGCTATATTTCTTTCGGGAAAAGTCACCAGACACTGATTGTTTACGCAGATTCGCCGGAGCGTCTGGTGAAGGACACCAATATTGTAACGACGACCCTGGAGGATTTAGGGCTGATCGTAACCTATTCAACCTTGAGTCTTGGCGCAGCGTATTTTGCTCAGTTACCTGGTAACTACACGTTACGGCCACGCCTGAGTTCTATCAGCAGTCTGAACTTTGCCGAAATGGAAAGCTTCCATAATTTCTTTACGGGCAAGGAGAAAGGAAATACCTGGGGTAACAACCTTATCACCCTGGGCGGCTCAGGAAATGATATTTATAACCTGAACTATCACATGACTACCGAGCATCAAAATTACTTCGGTAAAAACCCCACGCTGGGGCATACCGAAATTCTTGGTACGTCAAACGTGGGTAAAACGGTAGTGATGATGACTAAGGCTTTCGCCGCCCAGCAGTTCGGAACGCCGGAGTCTTTTCCACCTGAGAGGAAATTAAAGAAGTTAACTACAGTATTTTTCGACAAAGATCGGGCAGCGGAACCCGGTATACGTTCAATGGGAGGTGCTTACTTTCGGGTGAAAGAAGGAGAGCCGACTGGCTGGAACCCTGCGGCTTTGCCACCAACCAAACGAAATATTTCCTTTATGAAAGACCTGGTGAGGCTACTTTGTACGCTTAACAGTGAGCCGCTTGATGATTACCAGAACCGCCTGATTTCTGATGCCGTTGAGCGCCTGATGCAGAGGTCAAACCGCTCTTATCCGATCAGTAAGTTGCGACCACTCATTCTGGAGCCTGATGATACTGAAACCCGGCGGCATGGGCTTAAAGCACGCCTGAATGCCTGGGTGCAGGGAGGGGAATTTGGCTGGGTGTTTGATAACCCGGACGATACGTTTGATGTGGACAACCTGGACGTTTTCGGTATAGACGGAACGGAGTTTCTGGATAACAAAGTACTTTCCAGCGCCGCTTCATTCTATCTCATCTACCGTGTCACCATGCTGGCTGATGGCCGCCGACTCCTTATCTACATGGATGAGTTCTGGCAATGGATTAACAACGAAGCGTTCAGAGATTTTGTTTATAACAAACTCAAAACCGCGCGTAAGCTGGATATGGTGCTTGTCGTAGCGACACAATCGCCGGATGAACTAATTAAATCACCTATCGCGGCTGCCGTTCGTGAGCAGTGCGCAACACATATTTATCTGGCGAACCCCAAAGCGAAACGTAGTGAGTACGTAGACGAGTTGGAAGTGAGAGAACTTTATTTTGACAAAATTAAAGCCATTGACCCGTTATCTCGTCAGTTCCTGGTCGTCAAAAACCCACAGAGGAAGGGGGAAAGTGATGATTTTGCGGCTTTCGCAAGACTGGATTTGGGAAAAGCGGCGTATTACCTACCGGTTCTCAGCGCATCGAAACCCCAGCTTGAACTGTTCGATGAAATCTGGAAAGAGGGTATGAAGCCGGAAGAGTGGCTTGATACCTATCTGGAACGTGCAAACCTGATTTAAGGAATCCCTATGAAAAAGCATATCGTTGCGGCACTTATCGCCTCTGTTACGGTTATTTCTGGTGCTCAGGCTGGTGTTCCCGTTGCCATTGATGCAAACCCTGAATGGGCTGTCGAAGCGCAGCGCTGGACAGAACGCCTCAAACAGTGGCAGGACACCGTGAACCACTACCAGAAACAGATTAATGCCTATAAGCAGGAGCTGCTGACCAAAACGGGCATTCGTGATGTTCAGGGACTGGTGCAGTCTGCGCAATCTGTCAGTAAGGAGCTGGAGAATATTTACGACCAGGGGAACTCCTTCATTGATGATTACATTAAGAACCCGGAAGCCACCCTGTCTGAGCAAGCCAGATCACTACTGGCCGATTATAAGGAGAAAAACTGACCTGTAAGGGCTGGGATATACGGGCGACCTGGTGCGGGGCTGTGAAGCCTCGTTCCTGTCCCAACTGGCGGGGATCGAGTACGGCAATAAGCTGGAAAGTAAACTGCGTGAAGATAATCAGGAAATGGCCGACCTTATCGACCAGGTGAAGAACGCCCAGGACACGAAAGCCACGCAGGACGCGACAAACGCTGTATCTCTGGCAAGCCTGAAATTTAACAAGCTCAAATTTCAGTATGAAATGTATCGCGATAAGCAGCGCGATCTTGCAGAATACAAGGAGAAATGTTACAGGCTGCTTTCCAGGAGAAACAATTAGGGGCTGTTAATAAAGAAACTCCAGTTGTTGATTATAAGGCAGCATTTGAGCAGCAGAGTTAAAAAATGAACTAAGGGGGCATTTTGAAAAAATATTTTAGTTGCGGCGTTGGGGCTTACATTCTTTCTTACCGGGTGTGAAGAGGTTAAATCTGAGACTGGTGGCAGTCTCACGTGAAATGCAACGAAAAAAGTGCTGAATGCAAAAAGTCTGGGAGTGATAGCGAAAATTGTAAAAGGTAAGGAAGGTTTGTATTAAAGCAATGGCATTACGCAGCATAAAGATGCTTCAAGGTATTGGCTTTGGGTTAAGCCTGGAAGTTAAAGAAGCCATATGGTAAAACTTCTAGGGTTTCCGCTGGTACGTATTAATTACTTTTGCTATGGCTGATGGTATGCAGATACCAATCGTTGGGAAGTAAAAAATAGGATAAAGTGATGCTAAACAAATTGCATTAGCAAAATGTGATGGTATAATTTATTTGTAGGTCGGGACTGGTTAATTAGATGATTTTGTCAAAAATGGGCGGGAGGTTAAAATGAACAGGATGTTATCCTTTGCAGGCTTATAAACCTTTGAAAGAAAAAACACCTGCTCTGCATTTTGTCCAGCAGAAACTTATCTTTGTGTTCATTCTTTCGAAGTAAAAATATGCGGGGGTGGTTATGTTTATCAACGTCTGTAGTGAATTGTTGTCCGAGGAACTGACGGCGAAAAATTCCCGCAAGAAGCTATTATCTTTTGGTTTAATGAGTGTGGTTTAAGTAAATCAGATGTTATAATCGCAGTTTATTAACTGGAAGAATTTCGCATATACCCTTAAAGCTGGAGGAAGTTAAAATAAGCGATAATTAGAACTGCGGAGTTAATCAATTAGGGGTGGTTCAGGTTGTGGAGGCAATCTGATACCTGGCACTAATATCCCTCGCGCAGGGGTATAAATGTCGGAACGCATGTTAAAGCAATACAGACTGTTGATTGGGTGGGCGTCTCGGTTTTTCCCGTTGATGCCATTAAATCTGCTTTTATGAGTACTGGCATTGCGCATGAAAAGGCCTGGAAAAGATGGAAACAAAAGCTATGATGAAAACGGAGGTGTATGAAAAAGGCAGGAGTTTTTAGATTTTAGCCAGTGCGATAGATGCGGGGAGCTTCCGGTTTTCGCTTGGGTATTTAAAATGCAAAGGGGAAATTGTAAGGCACTCGACTAACGAGGAGGGTTAAGAGTCTGGAGTATACCGGTAACAGGGGAATCCCACCGGGCATCCGGAAAGAATGAGTAATTCCGCCGCTGGCTCCATGATGCCCGGCAGGGCATGGAAGGCGACACACCAGAGGCAGAATAAAATTCCCGTTCCTGAATCGCCGAAGCCCTCCCGTTTACGGGAGGTGCTTTTTTTGTATATTCCGTAAAACAGCGCTCGCGCCCCCTGCCAGGCCATTCGCGTAGCGAATTTATTTCCGCCCAAGTTAGCCGCGAATCCGTTGTCTCCGGTCGCAGACGCACCAGATCACAGCCACGATAGCCTTTCATTTTTTGCATTTCGATGGACGCTGAAGCACAATTTATGTGTGGAGGTAGTTCACTGCGATGCAGGATAGGGCAAACGCCGCAAAATGACGTCTCTGACGCCATTCCGCAACGTTTGCCCGGTGACCTACAGTCGGTTCTTGTTGGAGAGTTTTATGAAAAAAGTTCAATTCAGGATTGAAGAAAGCCAACATGATGATCTACTTGATTGTCTTAAAACACTTTATCCAGATGAACCGGGTTTAACCGTGGCAAAAGGAATGAAGCTGCTTGCCAATGCTTTACTGAAAAGCAAGGTTAAAAATGAAGATGTTAATGTGTCAAAAGACAATGATGATTTTATCAAAACCACAATGTATTTGACAGGGAAACAAAGAACTTTAATAGAGAAAGCGGCTCATCGTCATGGCTGGAATTTATCCAGAGAATGTCGTTATCGAATCCAGACGACACTTGAAAATGAACTTGATTTTTTCGATCAGGAACTTCTGGTGATGAATCGTTGCCGTAATGCAATAGATAAAATAGGGCGAAACTTTCATTATATTATCGTTAATGATAATGCCAGAGTACTTGATAAAGATGGGTTCTATCAGGACGCTGAGCGGTTGAGTTCAGAGATAATGAGTCTCAAAAGTGAGTTTGAAAACTACATTATGTTATGCAAAGGGCGAACTGTTTCTAACAAGGTTGAGGTGTAATCATGGGCGTTTATGTTGACAAGGAATTCAGGGTTAAGCGTAAGTCCTCTGAAGCTGGCCGCAAGTCTGCTTTCGCCCATAAAGTTAAAAATGGTGGTAAAAACTATCAACGCAACGTTCAGGAACGTATCAACCGCAAGGGTGCCAGTAAGGAGGTTGTTGTCAAAATCTCAGGAGGTGCGATTACCCGGCAGGGAGTTCGAAACAGTATTGACTATATGAGCCGGGAGTCAGAGCTGCCAGTGATGAGTGAAAGCGGTCAGGTATGGAAAGGTGATGAGATTCAGGAGGCTAAAGAGCACATGATAGATCGTGCTAATGACCCTCAAAATGTTTTCGATGATAAAGGTAAGGAAAATAAAAAGTAACCAAAATATTGTGTTTTCACCGCCTGTATCAGCAAAGGTAAAACCTGAAGATCTGCTTGAGTCTGTCAGGAAACGATGAACAAGAAGTACCCAATCATCGTTTTGTGCTTGGCTATCATAACGACAAAAAAGAACATCCACACGTGCATGTCGTTTTTCGTATTCGTGATAATGACGGTAAACGAGCTGATATCAGGAAAAAAGATTTACGGGAAATTCGTACAGGTTTTTGTGAAGAGCTGAAGATGAAGGGCTATGACGTTAAAGCCACTCATAAGCAGCAGCATGGACTTAACCAGTCTATTAAAGATGCGCACAAAACAGCGCCAAAACGACAGAAGGGCGTTTATGAGGTTGTTGATGTTGGCTACGACCATTATCAGAACGATAAAACCAAACCAAAGCAGCATTTCATAAAGCTTAAGACACTGAACAAGGGCGTGGAGAAAACATATTGGGGGCTGATTTTGGTGAGTTAACCACTCGCGAGAACGTTAAGAAAGGTGATCTTGTTAAGCTGAAGAAGTTAGGACAAAAGGAGGTGAAAATCCCTGCACTTGATAAGAACGGTGTACAGCATGGCTGGAAGACTGCACACAGGAATGAGTGGCAGCTAGAGAACCTGGGGGTTAAGGGCATAGACAGAATCTCTTCATCCAGTAAAGAGCTGGTGCTGAACAGCGCGGAGATGATTAAGAAACAGCAACTCCAGATGAGAAATTTCTCGCAAATAAAACAGTCGATGATTCAAAGCGAACAGAAAGTGAAAATCGGAATTCGTTTAGGATAATTCATACTCTCCGGTAACTTATTGATTAAGATCGATTTATTTATCTTGTTAGCAGAGTTTTTGTAAAAAGTAACTAGCGGTAAAAAATTTAGTTAACTTGACTATAAAATAAGCGCATGGTGATCAATCGATCTGCTTTTTTGTTATCAAATAGCATATGTATCTAGATATAAATAAAATACAAGCACAACAAAATGAAATGTGTCGTAAATGCGATCTAAAGATCTGCTTGTGTGTTGAACGCAGCACATGTAGCTGATAAAGTTTTTATGAGATGTAAATTTGTCCTTTTGTTTTTTTTCTCTGATTCGGGGTCATCCGAGTCGAGAGAGCGCCTGAAAGGAAAAACTATGGAATGGTATGTTTTGCAATTCACCACTACGAGATTTGCAGCAGTTTTTGCTCATCTCGAACGCCTGAACTTCTCTTACTTCTGTCCTATGGAGACTGAAAGGTATCGTCGCCCGGATAAGATAATTTCATATCGAGAAAGACGTTTGCCGCTTTTTCCCGGTTATCTTTTCCAGGCAGATTTTGAAGAGGTTCATTCCACTACAATAACTGCTATCCCTTACGTACAACGCTTTATCTCATTTGGCGGTGAGCCTTTACCTGTACCAGAAGATGTGATGGCGGAGCTTTTGTACAGACAATCACATACAACAGCTCAGGCAAATCTTTTAAGGAAATCAATTCCACACGATTTTGCTGAAATTCTACTCATGGATAATCCGCAGCAAAGAAGTATGGCTTTCATCCACTACATCACAGAGAGAAGCTTAACCCACAAAATGAAACGGAAGAAAAATGACTGCTACCCGAAAAAAAACCGCAACGAGACGCAAGCCCCAACGTAATACACTCTACCTGCCCTCAGCTTCTCGTGAGGAACTGGATAAAATTGCTCTGGAGATTGGCTATCGCAGGGGGCGACGTATTTCAAGCTCTGGTGTTGTCCAGTACCTCATAAAAAAATATTCCAGCCAGGCTATTGAGGAGCTGATAAACGAAGACGATGAAGACGATGACGACTTCGATGACTAACCATTCAGTCTAAACTTTCCGCGCTGTTCTGGCCGGGAGAAAATCATGTTATCTACTACTGCTTTTGCGGCGCTTGCTTTGCAATGCGCGGCCAGCGTTCATCCCGATACTGCGCATGAAGTTGCAAGGGTTGAATCTGGTTTTAACCCATATGCTATTGCCGAGATAATACCGAAGGTTGAACGTAAGCCCGGTGACAAAGGCGTAGTGTCCTACTTTCCCAAAACTAAGGAGGCTGCACTCCAGATCGTAAATCAAATTGAATCACGAAATCATCGTTACTCAGTCGGTCTTATGCAAATAACGAGTACGAATTTTGCAAACTTCAATACAACCGCTGAGAAAATGTTTGACCCCTGCGAAAATCTCAAGGTTTCAGAGCAAATTTTGGTGGATTGTTATAAACGTGGTGGCGATATTTTACGTGGCCTGAGTTGCTATTATTCCGGCAATCCAGAAACAGGAACTAAACCTGAATCAGATTTTAATAACACCAGTTATATACAGCGCATAGGGTTTAATCCGCCTGATAACAAAAAAACTGGGTGGTTCCTTCAGTTAAAGATGCAATCAGAAAGGAGAATGTAACACAGAGTATCAAACCTAAAGAAGTTACCGTATATCCCCAATATGCCATGCGTGGCACTGTTTTAGATGAAAAGGAAACAAACGATGTTAAAACTCAATAAACGTTATTTAACTCTTTCTGTATTTATGGCTGCATTGATGCTTTGTGTAGCAGAACCCGCCTTTGCTGATGATGTATCCACTAAAACAACTGGCTTCTTGCAGAAGATTATCGATTTTCTGACGGATATCCGTAAGCCAGCAATCACCATCATTGCGCTGGTAATTGGTTATATCGCTATCTTCTCACGCCAGCATGCAGCGTGGATTACGCCTTTAATTATCGGGATTATCATCTTTATTGTTGCGCCATATCTGCCTGACTGGTTAGCGTAATATGTAATAAAGGGGGCGTAAAAATGAGTACCGTTTTTAAAGGGCTGACACGCCCCGCTTTGATTAGGGGGCTGGGCGTTCCGCTCTACCCCTTTCTTGGAATGTGCGTTATCTGCGTTTTGCTTGGTGTCTGGATTCATGAGGCTATGTATGCCCTCATCCTCCCTGGCTGGTATGCCATCAAGCGAGTTACGAAGATAGATGAACGCTTTTTTGACCTGCTTTATCTGCGAATGCAAATCAAAGGTAATCCTCTGGCAAACAAGCGCTTCAATGCCGTCCATTATGCGGGGAGTTCGTACGACGCAGTCGATATATCGAAAGTGGACAATTTTATGAAGCTTAAAGACCAGTCTTCTCTTGAAGAGTTAATCCCGTATTCATCACACATCACTGATAACCTCATTGTTACCAGAAACCATGATCTACTGGCGACCTGGCAGATTGACGGAGCTTATTTTGAATGTGTAGATGAAGCGGATCTAGCCTTGCTTACTGACCAGCTCAATACGCTGATACGTAGTTTTGATGGGAAACCCGTTACCTTTTATACGCATCGTATTCGGGTTAGAAAAGAAGTGCGACCTGTATTTGACAGCAAGATCCCATTCGTCAACAGAGTGATGAACGATTATTACGAATCGCTTTCGGCGGCTGAGTATTTTGAAAACAAATTATACTTAACCGTATGCTATAAACCGTTCAGCGCTGAAGATAAAGTGACGCATTTTCTTTCAAGGAAAAAGGGTAATAAAATATCTTCGAAGAACCCATTAATGATATGAATGAAATATGTGGCAGATTGAGCACCTATCTCTCCCGGTTTCATTCCCGTCGTCTTGGGTTATATGAAGAAAATAATATAGTTTATTCAGAACAGCTTACGCTGTTCCAGAAACTGCTATCCGGGCGCTGGCAGAAAGTCAGAGTCACCAATAGTCCGTTTTATACATACCTGGGTGGTAAAGACCTGTTCTTTGGTAACGATGCTGGACAAATCACTGCCTCTGACCATGCCCGGTACTTTCGTTGCATCGAGATCAAGGACTACTTTCAGGAAACGGATGCCGGAATTTTTGATGCACTGATGTATCTCCCGGTTGAGTATGTACAGACTTCCTCTTTAACTCCGATAGACAAGCAGTCTGCAATCAAGGCGCTGGATGATCAGATCGACAAACTCGAAATGACCGATGATGCCGCTAAATCCTTGCTCGCTGATTTGAAAGTTGGTCTCGATATGGTCTCCAGCGGCTATATTTCTTTCGGGAAAAGTCACCAGACACTGATTGTTTACGCAGATTCGCCGGAGCGTCTGGTGAAGGACACCAATATTGTAACGACGACCCTGGAGGATTTAGGGCTGATCGTAACCTATTCAACCTTGAGTCTTGGCGCAGCGTATTTTGCTCAGTTACCTGGTAACTACACGTTACGGCCACGCCTGAGTTCTATCAGCAGTCTGAACTTTGCCGAAATGGAAAGCTTCCATAATTTCTTTACGGGCAAGGAGAAAGGAAATACCTGGGGTAACAACCTTATCACCCTGGGCGGCTCAGGAAATGATATTTATAACCTGAACTATCACATGACTACCGAGCATCAAAATTACTTCGGTAAAAACCCCACGCTGGGGCATACCGAAATTCTTGGTACGTCAAACGTGGGTAAAACGGTAGTGATGATGACTAAGGCTTTCGCCGCCCAGCAGTTCGGAACGCCGGAGTCTTTTCCACCTGAGAGGAAATTAAAGAAGTTAACTACAGTATTTTTCGACAAAGATCGGGCAGCGGAACCCGGTATACGTTCAATGGGAGGTGCTTACTTTCGGGTGAAAGAAGGAGAGCCGACTGGCTGGAACCCTGCGGCTTTGCCACCAACCAAACGAAATATTTCCTTTATGAAAGACCTGGTGAGGCTACTTTGTACGCTTAACAGTGAGCCGCTTGATGATTACCAGAACCGCCTGATTTCTGATGCCGTTGAGCGCCTGATGCAGAGGTCAAACCGCTCTTATCCGATCAGTAAGTTGCGACCACTCATTCTGGAGCCTGATGATACTGAAACCCGGCGGCATGGGCTTAAAGCACGCCTGAATGCCTGGGTGCAGGGAGGGGAATTTGGCTGGGTGTTTGATAACCCGGACGATACGTTTGATGTGGACAACCTGGACGTTTTCGGTATAGACGGAACGGAGTTTCTGGATAACAAAGTACTTTCCAGCGCCGCTTCATTCTATCTCATCTACCGTGTCACCATGCTGGCTGATGGCCGCCGACTCCTTATCTACATGGATGAGTTCTGGCAATGGATTAACAACGAAGCGTTCAGAGATTTTGTTTATAACAAACTCAAAACCGCGCGTAAGCTGGATATGGTGCTTGTCGTAGCGACACAATCGCCGGATGAACTAATTAAATCACCTATCGCGGCTGCCGTTCGTGAGCAGTGCGCAACACATATTATCTGGCGAACCCCAAAGCGAAACGTAGTGAGTACGTAGACGAGTTGGAAGTGAGAGAACTTTATTTTGACAAAATTAAAGCCATTGACCCGTTATCTCGTCAGTTCCTGGTCGTCAAAAACCCACAGAGGAAGGGGGAAAGTGATGATTTTGCGGCTTTCGCAAGACTGGATTTGGGAAAAGCGGCGTATTACCTACCGGTTCTCAGCGCATCGAAACCCCAGCTTGAACTGTTCGATGAAATCTGGAAAGAGGGTATGAAGCCGGAAGAGTGGCTTGATACCTATCTGGAACGTGCAAACCTGATTTAAGGAATCCCTATGAAAAAGCATATCGTTGCGGCACTTATCGCCTCTGTTACGGTTATTTCTGGTGCTCAGGCTGGTGTTCCCGTTGCCATTGATGCAAACCCTGAATGGGCTGTCGAAGCGCAGCGCTGGACAGAACGCCTCAAACAGTGGCAGGACACCGTGAACCACTACCAGAAACAGATTAATGCCTATAAGCAGGAGCTGCTGACCAAAACGGGCATTCGTGATGTTCAGGGACTGGTGCAGTCTGCGCAATCTGTCAGTAAGGAGCTGGAGAATATTTACGACCAGGGGAACTCCTTCATTGATGATTACATTAAGAACCCGGAAGCCACCCTGTCTGAGCAAGCCAGATCACTACTGGCCGATTATAAGGTGACAAACACCTGTAAGGGGCTGGGATATACGGGCGACCTGGTGCGGGGCTGTGAAGCCTCGTTCCTGTCCCAACTGGCGGGGATCGAGTACGGCAATAAGCTGGAAAGTAAACTGCGTGAAGATAATCAGGAAATGGCCGACCTTATCGACCAGGTGAAGAACGCCCAGGACACGAAAGCCACGCAGGACGCGACAAACGCTGTATCTCTGGCAAGCCTGAAATTTAACAAGCTCAAATTTCAGTATGAAATGTATCGCGATAAGCAGCGCGATCTTGCAGAATACAAGGAGAAAATGTTACAGGCTGCTTTCCAGGAGAAACAATTAGGGGCTGTTAATAAAGAAACTCCAGTTGTTGATTATAAGGCAGCATTTGAGCAGCAGAGTTATGAAATGAACTGAGGGGGCATTTTGAAAAAATATGGATTAGTTGCGGCGTTGGGGCTTACATTCTTTCTTACCGGGTGTGAAGAGGTTAAATCTGTAGACTGGTGGCAGTCTCACGTAGACGATGCAACGAAAAAAGTAGCTGAATGCAAAAAGTCTGGGAGTGATAGCGAAAATTGTAAAAACGCTAAGGAAGGTTTGTTTAGGTATAAGCAATTACATCCTAAGCATGTTGACTATAAAGATGCATTCAAAAATGTATTGGGTAAAGATGGAAGTTAAAAGGAGCCACTATGGCAACTTCTAGGTATTTCCAGGCCGCTCATGACGTTATTGTTAAAACTCTTGACCAGTCTTTGACTGGTCAGTTAGATAACTTTTCTTCAATAGCGTCTCAGTTGGGTAAGTATGGTATTTCCATTTATATATTGTGGTATGCTTATACCACAATAGTTGGGAAGCAAAAAACCGCAGTGCAGGATTTTTTATGGAATCTATGTCGATTCTGGTTAATTCTTGTTTTTGTCAAAATATGGGGGGGTGGTTAGATAGTGCAACTCAGGCCATTGATGGGCTGAAGGAAACATTTGCAGGAGGTGATCCTTGGAAATGGGTTGACGAGCTTTGGAGAAAGTTCAGCAGGTAGCTGCATATCTTATGTCCAAAGATACTTCGAAGTATGTGAAAACAGATGGGGCAATCGCATCTTTATTAACATATGCTGGAGGGATAATTGTATTATTGCTAACTTCAATAGTTTATCTCGCCGCCGAAGTTACTATTAAGATACTTACTATTACCGCGCCGTTGTTTATTATTTGTCTTTCATTTGGTTTTTTAAGGCAGATGTTTAATAGCTGGCTTCAGTTAATATTTAGCTCATGCTTCATATTCTTGTTTTGTGGACTGGCAATTAAAGCGGGAATGACGTTCCTTAATGGCATTCTGACAATCTCGATAGCTAACGCAGATGAATTAAATCTGATATCAACAGGTGCTCAGGCCGGGGTTGCAGGTGCATTTATGGCCTGGATTATCTGGCAAGCCAAAACCTATGCTTCCCAGCTCGCAGGGGTAGGTGTTGAAGGCGCGATGCAAGGGGCAGCCGCAATGGGGATTGGTGCTGGCGTGTTCGGCGCTTCCCGTATGGGGCGTAATCTGCTTGGTATGGGTAAAAATGCAGGGATTGGCGCATGGAAAGGCGTTCGCCGTCAGGATGGAGGGTTCAACCAGTCTCCTGGTGTATCCGGTAAGTTGGGGAATCTGACCGGGCAGGGCGTTAATATTGGCGCTAAGAGGCTTCGCCAGGCTGCGATAGATGCGGCGAAAAAGAAATATGGAGGTTAACCGATTTATTATTTAAATCTGTCAACTCTGAGTATTCCGCAGTAAATATTCACTCCTCTATTTAGGTTCTCACATTATGAAACTACTTATCACGGCTATCGTGACGCTTTGCCTTGTCGGATGCCAGGCGTCACATAAACAACCTCCGGTATCCGGAAAAAGCGAACCTGTTAATTCAGCAGAGGTAATGCGCAATGGGGTTTAAACTCTCAGGATTAAAACTTCCCGGTTTTAACAAGAAAAAAGAAGTAACAGACTCTTCAACTACTTTTGAAGAGAAAAAATGTATTGATGCAGGAGAAAATGAACCGCATTTATAAATTCGGTGGTATTGCAGGAATTGCCGTAGGTGTGTTGTCTCTGCTGGCACTGAATGGAGCGCTACCGTTAAAGAGTACGGTTGTCGAAGCTTATCTTATTAATGGTGTCACTGGCGTTGCTGAAAGACTGACTTCTGTTAAAAAAGAAAACCTCTCTGAAAATGAAGCGCTGGCGAAATACTTCATTACTCAGTATGTGAAGCGTCGTGAAGGGTATAACTATTTCAGTCTCCAGCATGATTATGATTATGTTCTGTTATACAGCGCTGAGAATGTGGCGACGGATTACAACGCTTTAATCAACGGCAACCAGTCACCAAAGGTTATTTATAATAAAGCGGAAAAAACCGCATCTGTTCAGGATAATCCGTCCGTGATTATCAGTCCGTCATCCCGGCGCGATGATAAGGATATGGGAGCATATATCCGGTTTAAATTAACCATTCGAAATGTCGCGACAGGCCAGGATGATTATGAATACTGGAACGTCCGGTTGACCTACCGTATTGAACCACAGGTCGAAATGGCATCGGGTGACCGAAACAATAACCCGCTTAAATTCGTTGTAACGAGCTATGTACGCGATAAAGAAGTGAAAGGGTGAAAACTATGAAAATGAATAAAGGAGCGCTAATTGTGGCGCTTCTGTTGGTGTCGCACGTCTGCCATGCAGCCGTTACGCCAGCAGCCAGCCGTTTTGATCCCCGTAATCAGATTGTCAGTTATAACCCGCAAAACACGACAGTCATAAACAGTGCGGTTGGCTATACAACCACACTGGTATTTGACGAGGATGAAACGGTCATCAGTGCCAGAACCGGCTTTCCCCAGGGCTGGGAAGTGAACAAAGAAGATAACCTGGTGTATCTCGAAGTGAAGCCCGTCAAACAGACCGTACAGAAGAACAGTACCGATGACAATGGCAACCAGTCTTCAGAGGTGGTCAGTGTAGCCCTTGATCCTGAAAACGACCTGGAACGCTGGCGAACAAACCTGTTTGTTCGCACAACAAAACGTAATTACAGCATGGAGCTGAACGCCCGAACGTTCCGGCAGCCGGATAAGATCGCGTTTGTTGTTAATTACCAGTACCCGCAGGAACGCCGGAAGGAGCAAGCCGAAATCGAGAAGAAACGGATTGAGGCTCTTAACAAGCGGCAGGAGGAGCAGGCAATCAACCGTTCTCTTGAAAATGCGAAGTCGCCCAAGAACTGGGATTACTGGAAGCGGGTAGCTGACGGCAGCCAGTCAATCAGCCCGGATTATGCGTATGACGATGGCCGCTATACCTGGTTTGGCTTCAGCCCCCTGAAGAAAATCCCCAGCGTCTTTGTTATGAGCGGCGAACAGGAAACGCTGACTAACCCGGTAGTCAAAAACAGCGGTAGTTTCACCGTTGTTGGTGTTCCTGTAGACCAGCGCTTTGTTTTGCGTATGGGAAATCAGGTTGTTGGGGTTGAAAACCGGGGCTTTGGCAAAGTCCGTCTTCCGGCAGGAGATACCGTTTCCCCGAACGTTGAGAAAGAGGTGATCCAGTGAGCGAACAGGAAAATAAAATCCCGACCGCTGTAGAAATTGAACAGGCACTACGGGAACGGCATCAGAAAGAAATTGAGCAAAACGATAAAACGGATAAGGGAGATGAAGATGACGGTAAATCAGTAAAACGCCTGGGTATCGAGAAGCTTAAAAATCGCGTAAGGGGCTGGTGATTATTGTGGCCGCTTTCCTTCTGCTGGCCGCCGGGGTGTCTGTCTACTATATCCCGTCGATTATTCGTGCAATGTCCAGCGGTGATGATAAGCCTGCCAGTAATGCGGTATCTACTGGTTCAGTAAAACGCGCGACAGGACTCAGCGATGATGTTGATCCATTCAACACCAGGGAAGAACCGGCTAAGACGGAAGAACGGAAGGAAAGCAGCGGCAAAGCGGAAACGCCACCGGAAAAAGTACAACAAAACTTCAGTCGTGCGCTGGATGTGGCCTATGGGGGGAGCAGCTCTGCTTCCTCAAATTCCGGTGGTAGCTCATCAACGGCCAGCAATACCAGGAACGAGGGGGACGGCAGTGACAAACAGGCAGAGGTTCAGCCTGTTAATGCAGGGCAACCCGCGCAGCTTTCTAAAATTACGCGCGTTCCTTATGACCCTAACCTTTTCATTCCTGAAAATACCGCGATTAAGTGCTCGCTGGACAGACGATTTATTTCTGATCTGGCTGGCAAGCTGGTCTGTACGATTAATGAGGACGTATACAGTGCAAACAGGAATGTAAAGCTAATTGAAAAGGGAACAGCCGCATATCTGATGTATAAAACGGGCACGTTCAATCATGGTCAGGGAGCCGTGTTTATCGCTGCTACGAAGCTTAGAACACGTAAGGAACCTTTCATTGATATTCCGCTGATTGATACACAGGCGGCGGGAGCGCTGGGTGAGGCCGGGGCTTCAGGCTGGATTGACACACATTTTGCAGATCGCTTTATGGGCGCGATGATGGTTGGCATGATCCCCGATGTGGCACAGGCCGCAAGTGGAGCGGCTAAAAGTAATAAAGACAATCAGACCGACTATACAGCTAACAGTCGCCAGGCTTTTGCTGATATAGCACGCGAAGCGTTTGCAAACAGCGTAAATATCCCTCCAACGCTTTATAAAAACCAGGGGGAAATCATTACTCTGATCGTTGGTCAGGATTTGGATTTCTCCAGCATCTACAAATTAAAATGGTGGGTACCCGGAGGTAATTTGATAACGCTGGATATCAAAGAGTTTTCAATGCTGTTAGGAATAAGGGAAAGCGAAATATACCATCATATTCGTAAGGGCATACCAATAAATGGCGTTCCTTTCCCCAAACCACTGAAACAAATCAAAACGCATCGATTTAATTACGAAGAAGTCATGCGTTTTATTGAAGATCTTAAAGGAAAGGTGAATTATGAAGGATGCAGAGAACAGACATATTGTTTATGAAATAATAGATGAATACTCCACCACTGGCTTAATGAAGTCGATGGTTTAACTGAAATCGCGGTAAATCGGCCTGATGAACTGTTTGTTAAAGTGAGTGGGAAATGGCAGCAGCATGAATTAAAAATGGATTTTAAAGATTGTATGTCATTTGCAGGGGCAATTTCTGATTATCATGATGGTGGGTCGGTTACGCCAGAATATCCCTTACGTTCTGTTACGCTCCCCGGAGGGGAGCGTGTACAAATAGTTATTCCCCCGGCAACGGAACGGGAAACGGTGTCTATTACAATCCGTAAGCCGTCCAGTGTGTTTATCGACCATGATACCTTTGTTAAACAAGGGTTTTACTCAAGGCTAAATCAAGGGGGGAAATTCAGGGATAAAAGAATGAATTATCTTCAATGTTTAAAGATAATTGTTTTGAACAATTTGTCCCTGAATGCCTGGTTAAGGGTAAAACGATGGTATTTTGTGCCGGAACGGGGGCGGGTAAAACCACCTTTGCAAATGCCTGTCTTCAGTACATACCCCATCATTTACGATGTATTTCAATTGAAGATACAGATGAAGCAAAGTTCAGGTTCCACAAAAATCATGTGAAATTGTATTACCCGTCAGAAGGGGAAAGTAGCACTGTCAGTTCCGCTTCTCTTTTACGTTCTGGTTTTCGTATGAACCCGGACAGAATCCTCATGACTGAGGTTCGCGGCGCGGAAGCCTGGGATTTTCTCAAAGGCTCAAGTTCCGGCCATGCAGGGAACCTTACAACCGTCCATGAGAGCACCCCTGAAGATGCTGTGCTTGGACTGGTTCAACGGTGCTATATGAACCCTGAATGTCAGAATTTGCCTTATAACATTATATTGCGGAGGGTGTTAAGCAACGTCGATGTGATTATGAGTATTAAGTACATCGATGAGGAAGATAACCGATTTGCATCGGGTATTTACTATCGTGATATTCATTTTCAGGAATACTTTGAAAAGCTGAAGGAGTGATTATGTCTCTGAAACTACCCGATAAAGGCCAGTGGGCTTTTATCGGTCTGGTTATGTGTCTTGTGACGTATTATACTGGCTCAGTTGCTGTTTACTTCCTGAACGGAAAAACGCCGCTTTATATATGGAAGAATTTTGATTCCATGCTGCTGTGGCGGATAATAACAGAGTAATATACGGACAGATATCAGGTTAACCGCAATCCCCTCTTTTTATCAGGTATGGTTTCGTCTCTCATTGTGCCTGTTTTTATTATCTGGCAACTGAATAAAACGGATGTTGCTCTTTATGGTGACGCGAAGTTTGCCAGTGATAATGATTTAAGAAAATCGAAACTTCTGAAATGGGAGAAAGAAAACGATACTGATATTCTTGTAGGTGCATATAAAGGTAAATACCTGTGGTATACCGCACCAGATTTTGTATCACTTGGCGCAGGAACCCGCGCAGGTAAAGGGGCTGCAATAGGTATTCCAAACCTGCTGGTCAGGAAACATTCCTGATTGCATTAGATCCAAACAGGAACTGTGGAAAATCACCAGTAAGGTACGTGAAAAACTGCTGGGTAATAAGGTCTATCTTCTTGACCCTTTCAACAGTAAAACGCATCAGTTTAACCCGCTTTTCTATATTGATTTAAAAGAAGAGAGCGGGGCTAAAGACCTGCTGAAGCTGATTGAGATCCTGTTCCCGTCTTACGGTCTGACCGAGCGGAAGCACACTTTAATAACCTCGCGGGGCAGTACTGGACGGGGCTGGCAAAATTGCTTCATTTCTTTATTAACTACGACCCCTCCTGGCTTGGTGAGTTCGGGCTTAAACCTGTTTCTCAATTGGTTCTGTAGTCGATTTGTACAGCAACATCGACCGGGAGCTGATACTCAGTAAGCGAGAAGACCTGGAGGGAACAAAAGGACTTGATGAAAACGCGCTGTATCATCTGCGCGATGCCCTGACCAAAATCAGGGAGTATCACGAAACCGAAGACGAACAACGTTCGAGTATTGATGGTTCGTTTCGTAAGAAAATGAGTCTGTTTTATCTTCCGACTGTTCGCAAATGTACAGACGGCAATGATTTCGACCTTCGCCAGTTGCGCCGGGAAGATATCACTGTTATGTTGGCGTTAATGCTGAAGATATGTCCCTGGCCTATGACTTCCTCAACCTGTTCTTTAACTTCGTTGTGGAAGTCACGTACGAGAAAACCCTGACTTTGACCCGACACTGAAAC
